# Proteomic profiling of human plasma extracellular vesicles identifies PF4 and C1R as novel biomarker in sarcopenia

**DOI:** 10.1002/jcsm.13539

**Published:** 2024-07-15

**Authors:** Paula Aparicio, David Navarrete‐Villanueva, Alba Gómez‐Cabello, Tresa López‐Royo, Enrique Santamaría, Joaquín Fernández‐Irigoyen, Karina Ausín, Manuel Arruebo, Victor Sebastian, Germán Vicente‐Rodríguez, Rosario Osta, Raquel Manzano

**Affiliations:** ^1^ LAGENBIO Laboratory, Faculty of Veterinary University of Zaragoza Zaragoza Spain; ^2^ Centre for Biomedical Research in Neurodegenerative Diseases (CIBERNED) Instituto de Salud Carlos III Madrid Spain; ^3^ AgroFood Institute of Aragon (IA2) Zaragoza Spain; ^4^ Aragon Health Research Institute (IIS Aragon) Zaragoza Spain; ^5^ EXER‐GENUD (Growth, Exercise, Nutrition and Development) Research Group University of Zaragoza Zaragoza Spain; ^6^ Faculty of Health Science University of Zaragoza Zaragoza Spain; ^7^ Defense University Center Zaragoza Spain; ^8^ Proteomics Platform, Clinical Neuroproteomics Unit, Navarrabiomed Hospital Universitario de Navarra (HUN), Instituto de Investigación Sanitaria de Navarra (IdiSNA) Pamplona Spain; ^9^ Instituto de Nanociencia y Materiales de Aragón (INMA) CSIC‐Universidad de Zaragoza Zaragoza Spain; ^10^ Department of Chemical and Environmental Engineering University of Zaragoza, Campus Río Ebro‐Edificio I+D Zaragoza Spain; ^11^ Networking Research Center on Bioengineering, Biomaterials and Nanomedicine CIBER‐BBN Madrid Spain; ^12^ Centro de Investigación Biomédica en Red de Fisiopatología de la Obesidad y Nutrición (CIBERObn) Madrid Spain; ^13^ Faculty of Health and Sport Science (FCSD), Department of Physiatry and Nursing University of Zaragoza Zaragoza Spain

**Keywords:** Ageing, Sarcopenia, Extracellular vesicles, Exosomes, Biomarkers, Plasma

## Abstract

**Background:**

Sarcopenia, the gradual and generalized loss of muscle mass and function with ageing, is one of the major health problems in older adults, given its high prevalence and substantial socioeconomic implications. Despite the extensive efforts to reach consensus on definition and diagnostic tests and cut‐offs for sarcopenia, there is an urgent and unmet need for non‐invasive, specific and sensitive biomarkers for the disease. Extracellular vesicles (EVs) are present in different biofluids including plasma, whose cargo reflects cellular physiology. This work analysed EV proteome in sarcopenia and robust patients in the search for differentially contained proteins that can be used to diagnose the disease.

**Methods:**

Plasma small EVs (sEVs) from a total of 29 robust controls (aged 73.4 ± 5.6 years; 11 men and 18 women) and 49 sarcopenic patients (aged 82.3 ± 5.4 years; 15 men and 34 women) aged 65 years and older were isolated and their cargo was analysed by proteomics. Proteins whose concentration in sEVs was different between sarcopenic and robust patients were further validated using ELISA. The concentration of these candidates was correlated to the EWGSOP2 sarcopenia tests for low muscle strength and low physical performance, and receiver operating characteristic (ROC) curve analyses were carried out to evaluate their diagnostic power, sensitivity and specificity.

**Results:**

Proteomic analysis identified 157 sEVs proteins in both sarcopenic and robust samples. Among them, 48 proteins had never been reported in the ExoCarta nor Vesiclepedia databases. Statistical analysis revealed eight proteins whose concentration was significantly different between groups: PF4 (log2 FC = 4.806), OIT3 (log2 FC = −1.161), MMRN1 (log2 FC = −1.982), MASP1 (log2 FC = −0.627), C1R (log2 FC = 1.830), SVEP1 (log2 FC = 1.295), VCAN (FC = 0.937) and SPTB (log2 FC = 1.243). Among them, platelet factor 4 (PF4) showed the lowest concentration while Complement C1r subcomponent (C1R) increased the most in sarcopenic patients, being these results confirmed by ELISA (*P* = 1.07E‐09 and *P* = 0.001287, respectively). The concentrations of candidate proteins significantly correlated with EWGSOP2 tests currently used. ROC curve analysis showed an area under the curve of 0.8921 and 0.7476 for PF4 and C1R, respectively. Choosing the optimal for PF4, 80% sensitivity and 85.71% specificity was reached while the optimal cut‐off value of C1R would allow sarcopenia diagnosis with 75% sensitivity and 66.67% specificity.

**Conclusions:**

Our results support the determination of EV PF4 and C1R as plasma diagnostic biomarkers in sarcopenia and open the door to investigate the role of the content of these vesicles in the pathogeny of the disease.

## Introduction

Sarcopenia is defined as the progressive loss of muscle mass and function usually associated to ageing and represents an important public health concern given the rising life expectancy. Sarcopenia is a risk factor for frailty and, therefore, for dependency, disability and mortality.^1^ Beyond demographic and social impact, sarcopenia represents an economic burden due to the associated need for hospitalizations and healthcare expenditure [S1], which has been calculated in 2019 as 40.4 billion USD in the USA.^2^ Strategies to diagnose, prevent and reverse this condition are thus in great demand.

Different international groups have developed their own definition and diagnostic criteria, tests and thresholds for the disease.^3^, ^4^, ^5^, ^6^, ^7^ These documents have represented an undoubted advance in the field since they have established recommendations on clinical and imaging parameters, physical function tests and gender specific cut‐off points together with an algorithm for the search, confirmation and analysis of the severity of cases, especially the document published by European Working Group on Sarcopenia in Older People (EWGSOP) in 2010, which was a milestone for sarcopenia in Europe. However, still without any findings on molecular biomarkers that could help in the determination of the disease, all these diagnostic guidelines are based solely on physical parameters, with the consequent problems of repeatability, availability or training that they entail. As a result, and despite the above‐mentioned efforts, there is a current disparity in diagnostic tests and cut‐off values, which may have had a significant influence on epidemiology and have contributed to under‐diagnosis and delayed treatment of the disease to date.^8^ The development and validation of a panel of multiple biomarkers, including serum and tissue markers, that reflect pathophysiological processes directly and/or indirectly linked to muscle ageing, could solve the urgent need for specific, non‐invasive, easy and cost‐effective tests to facilitate identification and diagnosis of sarcopenia and monitor the effectiveness of treatment in a wide variety of clinical settings and in different patient populations.

Extracellular vesicles (EVs) are membrane‐enclosed vesicles secreted and released into the extracellular medium (including the bloodstream) by most cell populations. According to MISEV2023 guidelines, unless researchers can establish the specific biogenesis pathway of the vesicle, authors are urged to consider use of operational terms for EV subtypes that refer to physical characteristics of EVs, such as size comprising small EVs (sEVs, <200 nm) and large EVs (lEVs, >200 nm).^9^ They have a lipid membrane and contain different biological molecules such as proteins, nucleic acids such as microRNAs, or lipids, among others [S2]. These vesicles act as signalling pathways when captured by receptor cells, forming a new and complex intercellular communication system, which has been shown to play relevant roles in processes such as proliferation, inflammation or neurodegeneration. Although the composition and concentration of EV cargo change depending on cellular conditions and physiological state and vary in the course of a pathology, it is important to note that EV cargo is actively loaded and may differ from molecules and concentrations present in the non‐EV medium. Therefore, EV cargo does not necessarily represent total media composition.^10^, ^11^ In fact, studies have already shown that EVs may be beneficial in the investigation of diseases such as amyotrophic lateral sclerosis (ALS) or Parkinson's disease among others.^12^, ^13^

In the musculoskeletal system, EVs have been shown to actively participate in stem cell‐mediated myogenesis, providing biochemical signals important for muscle regeneration, protein synthesis and hypertrophy.^14^, ^15^ Accordingly, EV concentrations rise during exercise, which may indicate a relevant role in exercise‐related signalling mechanisms and tissue crosstalk.^16^ From a therapeutic perspective, recent studies have demonstrated the efficacy of EVs restoring muscle cell membrane integrity in dystrophic mice.^17^ Even if the underlying molecular mechanisms remain to be elucidated, these studies highlight the relevance of EVs in the pathophysiology of skeletal muscle disorders and open the field to the use of these vesicles as biomarkers and therapeutic drugs vehicles to treat these conditions, including sarcopenia.^18^, ^19^

The extraction and purification of EV has been optimized over the last few years, and there are available systems to efficiently isolate EVs from small volumes (below 500 μL) of different starting samples, which makes them ideal for diagnostic purposes. In this line, two test kits based on liquid biopsy approach are commercially available for rapid detection of a gene panel (ExoDx®‐IntelliScore [S3]) and EML4‐ALK molecule (ExoDx® Lung [S4]) in urine and plasma exosomes for prostate and lung cancers respectively.

In this research, we sought to define the EV proteomic signature of sarcopenic patients and look for differentially regulated proteins, compared with robust individuals that can be used as potential biomarkers in the diagnosis of this disease. Here, we performed proteomic analysis of circulating sEVs in healthy controls and sarcopenic samples and identified, for the first time, eight differentially expressed proteins between groups.

## Materials and methods

### Patient selection

**Figure 1:**
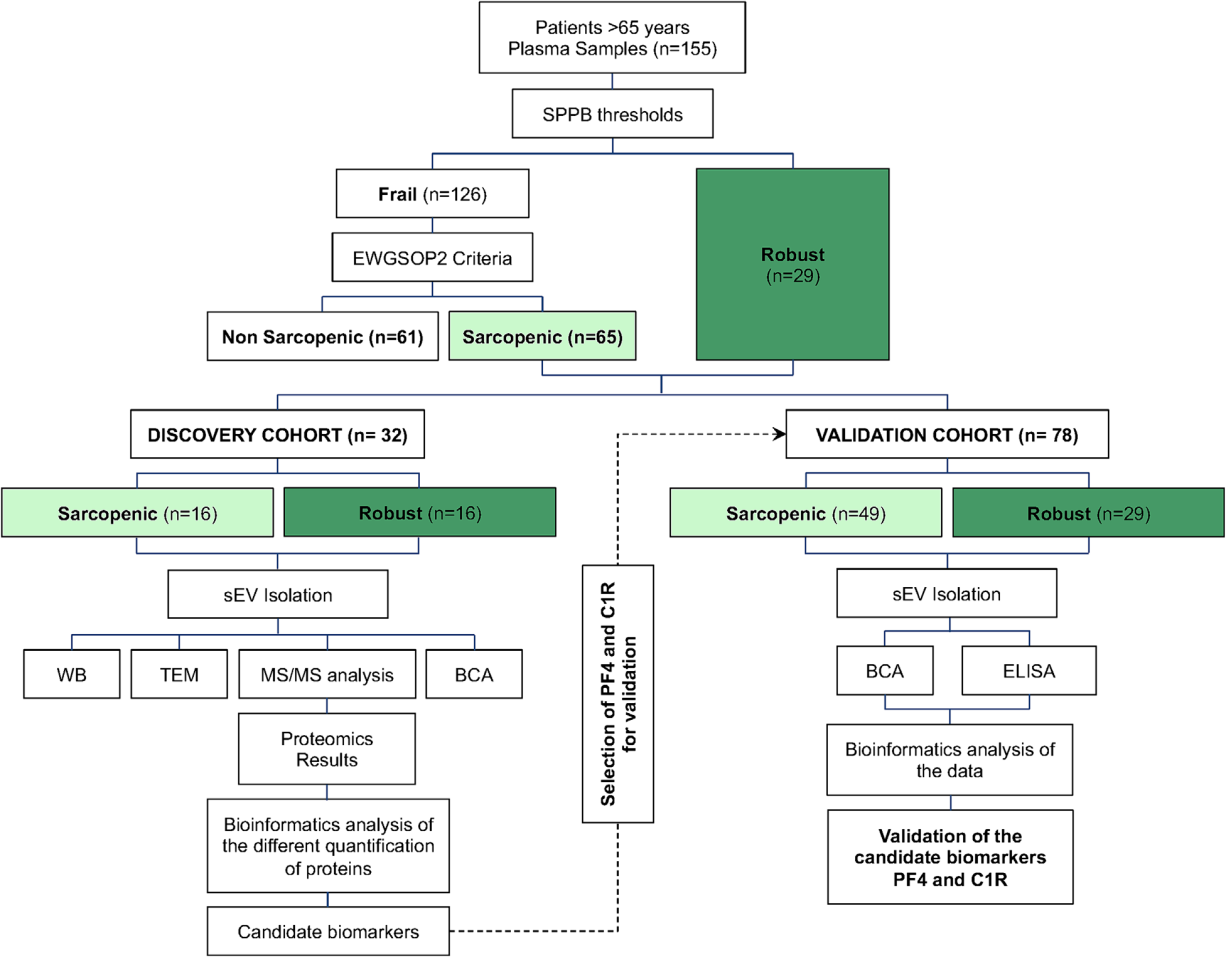
Schematic workflow of the study. BCA, Bicinchoninic acid assay; C1R, complement C1r subcomponent; ELISA, enzyme‐linked immunosorbent assay; EWGSOP, European Working Group on Sarcopenia in Older People; PF4, platelet factor 4; SEM, scanning electron microscopy; SPPB, Short Physical Performance Battery; TEM, transmission electron microscopy; WB, western blot.

In brief, an *initial cohort* of 155 participants over 65 years and four health centres from Zaragoza, Spain, within the framework of the EXERNET‐Elder 3.0 project.^20^ Data were registered in the electronic repository clinicaltrials.gov (reference number: NCT03831841). A total of 155 participants were screened and classified from a functional perspective as frail (*n* = 126) or robust (*n* = 29) according to the *Short Physical Performance Battery* (*SPPB*) thresholds (frail ≤ 10 points; robust > 10 points) [S5]. Operational criteria and cut‐off points proposed by the EWGSOP2^4^ were used to determine the sarcopenic patients among the participants. Specifically, *Grip strength* and *Chair stand* for low muscle strength and *Gait speed*, Time up and go 3 m (*TUG 3 m*) and *SPPB* for low physical performance were used to detect sarcopenia and low muscle mass was assessed among the screened frail cohort. In this sense, Bioelectrical Impedance Analysis (BIA) and body composition analyser (TANITA BC418MA, TanitaCorp) was used to measure body weight (kg) and estimate appendicular skeletal muscle (ASM; kg) and SMI (Skeletal Muscle Mass Index; ASM/height^2^) using the formula proposed by Janssen *et al*. in 2004 [S6]. Each criteria were considered as positive when, at least, one of the cut off tests was unmet (*Table* 1). Following these criteria, 65 frail patients were diagnosed as sarcopenic and 61 as non‐sarcopenic frail (*Figure* 1). Once diagnosed as robust, frail sarcopenic and non‐sarcopenic frail, the patients were separated in two cohorts (*Figure* 1). For the *discovery cohort* (*n* = 32), 16 older adults were randomly selected from sarcopenic patients (8 men and 8 women) and robust controls (8 men and 8 women). For the *validation cohort* (*n = 78*), 33 sarcopenic patients (mean age 82.3 ± 5.4 years; 7 men and 26 women) and 13 robust controls (mean age 73.5 ± 5.6 years; 3 men and 10 women) were selected from the initial cohort and analysed together with the patient samples from the *discovery cohort*.

**Table 1:** Demographics and characteristics of selected samples.

	Discovery cohort			Validation cohort		
Descriptive data	Robust (*n* = 16)	Sarcopenic (*n* = 16)	*P value*	Robust (*n* = 29)	Sarcopenic (*n* = 49)	*P value*
Sex [*n* (%)]						
Male	8 (50)	8 (50)		11 (37.9)	15 (30.6)	
Female	8 (50)	8 (50)		18 (62.1)	34 (69.4)	
Grip strength (kg)^a^	30.39 ± 2.44	17.47 ± 1.68	0.000175***	26.77 ± 1.65	18.57 ± 1.23	1.75E‐05****
Chair stand (s)^a^	9.33 ± 0.35	24.29 ± 1.82	7.40E‐07****	10.42 ± 0.43	21.59 ± 0.77	1.00E‐15****
SPPB (points)^b^	11.80 ± 0.11	5.88 ± 0.41	3.33E‐09****	10.97 ± 0.24	6.65 ± 0.23	1.00E‐15****
Gait speed (m/s)^b^	1.30 ± 0.03	0.51 ± 0.05	1.40E‐14****	1.18 ± 0.05	0.60 ± 0.02	1.00E‐15****
TUG 3 m (s)^b^	5.91 ± 0.08	17.00 ± 2.14	3.00E‐08****	7.21 ± 0.34	12.77 ± 0.85	1.11E‐10****

*Note*: Data are shown as mean ± *SEM*. Descriptive sarcopenic patients and healthy robust controls in discovery cohort (*n* = 32) and validation cohort (*n* = 78).

Abbreviations: SPPB, Short Physical Performance Battery; TUG, Time Up and Go 3 m.

^a^ Differences between robust and sarcopenic groups for continuous variables determined by Student's *t*‐test for normally distributed data. Statistically significant difference at *P* < 0.05.

^b^ Differences between robust and sarcopenic groups for continuous variables determined by Mann–Whitney *U* test for non‐normally distributed data; statistically significant difference at *P* < 0.05.

*P < 0.05, **P < 0.01, ***P < 0.001, and ****P < 0.0001.

### Isolation and purification of small extracellular vesicles from human plasma

Fasting blood samples (3 mL) were collected by venipuncture from all the participants in heparin‐containing tubes, processed immediately and stored at −80°C. Participants were advised to avoid intense physical exercise on the day prior to blood sample extraction. Plasma samples were thawed at 4°C and size exclusion chromatography was performed following the manufacturer's protocol (PURE‐EVs: size exclusion chromatography columns, HBM‐PEV, HansaBiomed). EV‐enriched fractions were pooled and subjected to a two‐step centrifugation to concentrate the EVs and further remove small plasma proteins improving sample purity: (1) Vivaspin6 300‐kDa MWCO, VS0651, Sartorius; (2) 10‐kDa MWCO filter Amicon Ultra‐0.5, UFC501096, Merck Millipore. The supernatant containing the purified EVs was collected and stored at −80°C until further processed. More information for blood collection and EV isolation is detailed in *Supplementary Information*.

### BCA protein assay

Prior to freezing the samples, protein concentrations of the EV‐enriched fractions were measured by micro bicinchoninic acid (BCA) protein assay using a bovine serum albumin (BSA) serial dilution as standard curve according to the manufacturer's instructions (23235, ThermoScientific). Of isolated EVs from each patient, 5 μL were pipetted in duplicate into 96‐well plates and mixed with BCA reagents up to 300 μL. The covered plate was incubated at 37°C for 2 h, and absorbance was measured at 560 nm on a TECAN Infinite 200 plate reader. Absorbance values were extrapolated from the standard curve using a best‐fit polynomial equation, with *r* ^2^ > 0.99 for each assay.

### Electron microscopy

#### Transmission electron microscopy (tem)

In order to define the resulting particle size, EVs were measured using transmission electron microscopy (TEM) (Tecnai T20, FEI Company). Organic staining of the EV dilution was carried out using phosphotungstic acid dissolved in Milli‐Q water (30 mg/mL) mixed at a 1:1 (v:v) ratio with the sample and incubated for 45 min. After washing to remove the phosphotungstic acid excess, samples were placed on TEM grids (carbon fibre copper microscopy stand, Electron Microscopy Sciences FCF200‐Cu). Images were captured by a FEI Tecnai T20 microscope at different magnifications and processed using ImageJ to determine the Minimum Feret Diameter of each vesicle. At least 100 vesicles were measured from each sample. Results were represented as diameter size distributions and mean diameter ± *SD*.

#### Scanning electron microscopy

EV morphology was observed by a CSEM‐FEG Inspect 50 field‐emission scanning electron microscope (SEM) (FEI Company) at high vacuum and with an acceleration voltage of 20 kV. For sample preparation, a drop of the corresponding resulting EV dilution was placed on a slide, fixed with carbon tape to an SEM microscope holder, air‐dried and sputtered with palladium to promote electron conduction.

### EV size and concentration

The size distribution and concentration of the EVs were determined by nanoparticle tracking analysis (NTA) with a ZetaView instrument (Particle Metrix). Samples were diluted 500 times in PBS to achieve a particle concentration within the optimal range for analysis reported by the manufacturer.

### Western blot

A volume of EVs corresponding to 15 μg of total protein was lysed in Laemmli buffer and heated at 95°C for 5 min for all the test performed. Proteins were separated through a 12.5% sodium dodecyl sulfate‐ polyacrylamide gel electrophoresis (SDS‐PAGE) gel and transferred to a PVDF membrane (Amersham™, GE Healthcare Life Sciences). Membranes were sequentially blocked with a Tris‐buffered saline solution containing 5% skimmed milk and 0.1% Tween for 1 h at room temperature and incubated overnight at 4°C with antibodies against conventional EV markers: CD81, CD9, ALIX and HSC70 and non‐vesicular protein CALNEXIN. Then membranes were washed with *tris*‐buffered saline with 0.1% Tween20, incubated with horseradish peroxidase‐conjugated anti‐mouse or anti‐goat secondary antibodies and washed again to remove unbound antibody. Finally, chemiluminescence detection was performed using Immobilon Crescendo Western HRP Substrate (Millipore) in a Molecular Imager® VersaDoc™ MP 4,000 system. Extended details regarding the antibodies can be found in *Supplementary Information*.

### Protein identification by mass spectrometry

Proteomic analysis of EV was performed simultaneously by using nanoscale chromatography coupled online to the Orbitrap‐XL (Thermo) mass spectrometry. Proteins were identified according to the Uniprot Reference Proteome database with March 2021 release. The Perseus software (version 1.6.14.0) was used for statistical analysis and data visualization. Extended details regarding this section can be found in *Supplementary Information*.

### ELISA

PF4 and C1R concentrations in EVs were quantified using a human PF4 DuoSet ELISA kit (DY795, R&D Systems) and Human C1R ELISA kit (ELH‐C1R, RayBiotech), respectively, according to the manufacturer's instructions. The standards and the samples were run in duplicate. Results were read using a TECAN Infinite 200 plate reader. PF4 and C1R concentrations were normalized to total EV protein content using the BCA protein assay kit (Pierce, Thermo Fisher Scientific) in parallel to the ELISA assays.

### Statistical analysis

Results were statistically analysed using GraphPad Prism 9 statistical software. Outliers were detected by iterative Grubb's test and excluded from the analysis. For comparisons between two groups, parametric Student t test or nonparametric Mann–Whitney test were used. Two‐way univariate analysis of variance (ANOVA) and Bonferroni post‐hoc tests were performed to identify the effect of the disease (sarcopenic/robust), sex (men/women) in the concentrations of these proteins and/or the age of patients. Metascape (http://metascape.org/gp/index.html#/main/step1) and STRING (version 12.0) [S7] were used to identify and plot enriched GO terms and protein–protein interaction networks. The online tool (http://bioinformatics.psb.ugent.be/webtools/Venn/) was used to construct the Venn diagram. Spearman rank correlation tests and simple linear regression were performed to examine the relationship between candidate protein levels and the scores of the main sarcopenia diagnostic tests. To evaluate the diagnostic accuracy of the two proteins validated, we carried out a receiver operating characteristic (ROC) curve analysis using the Wilson/Brown method. The area under the curve (AUC) was then estimated with a 95% confidence interval (CI). Data are shown as mean ± standard error of the mean (*SEM*) unless otherwise specified. A significance threshold of 0.05 was selected for statistical testing and statistical significances were as follows: *P* ≤ 0.05 (*), *P* ≤ 0.01 (**), *P* ≤ 0.001 (***) and *P* ≤ 0.0001 (****) compared with robust controls.

## Results

### Characterization of plasma EVs isolated by SEC

**Figure 2:**
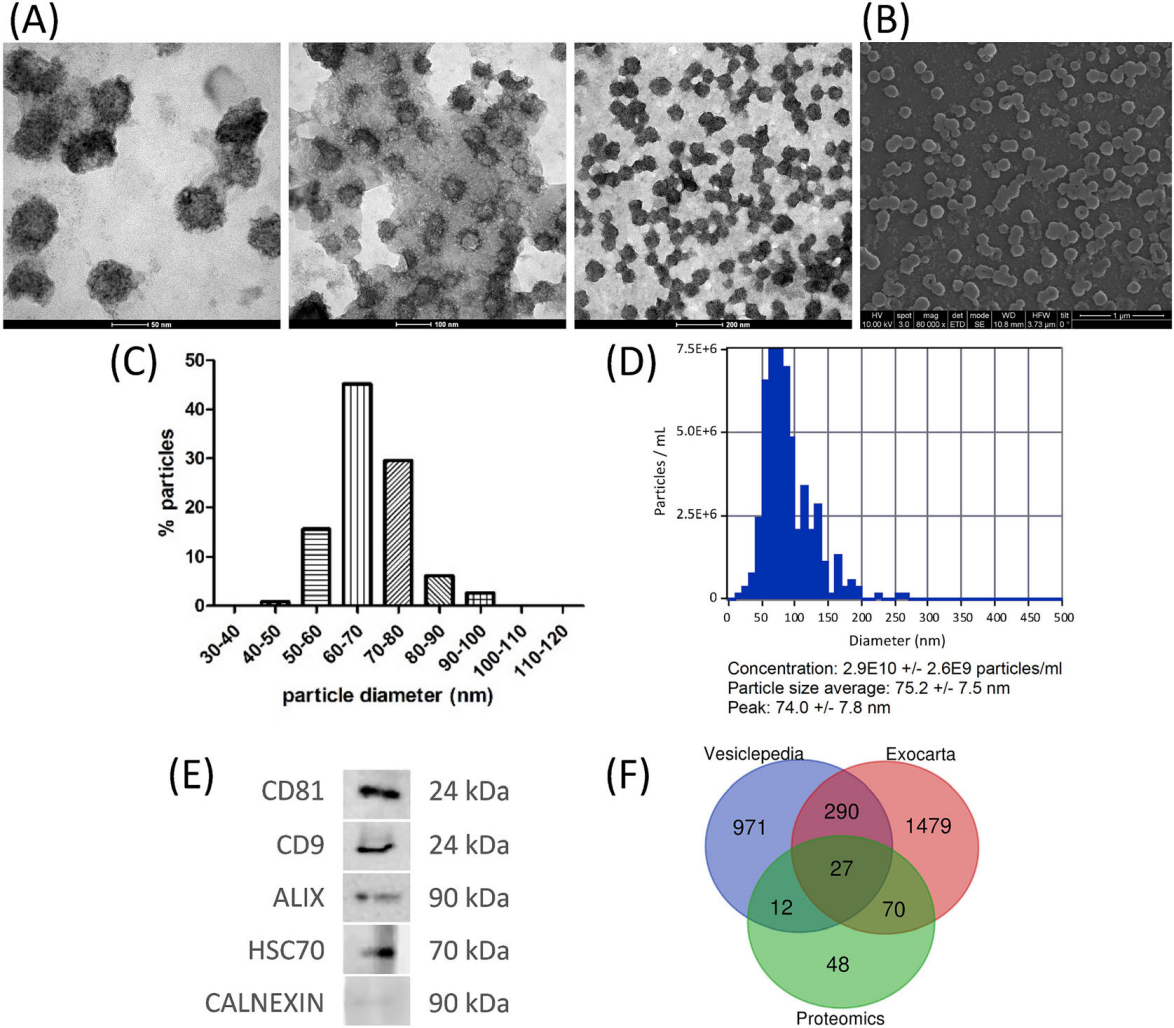
Characterization of plasma small extracellular vesicles (sEVs) isolated by size exclusion chromatography. (*A*) Transmission electron microscopy (TEM) and (*B*) scanning electron microscopy (SEM) images showing the size of the sEVm and their typical circular and cup‐shaped morphology (scale bars of 50 nm, 100 nm, 200 nm and 1 μm, from left to right). (*C*) Histogram of the particle minimum Feret's diameter of sEVm as measured from SEM images. *N* > 100 particles were individually measured. (*D*) Representative nanoparticle tracking analysis (NTA) profile of sEVs isolated. (*E*) Representative Western blots indicating that the isolated sEVs are positive for the EV markers CD81, CD9, ALIX and HSP70 (*F*) Venn diagram describing the overlap of proteins identified in present study with those published in the Vesiclepedia and ExoCarta database.

Transmission (*Figure* 2A) and scanning (*Figure* 2B) electron microscopy identified a large population of cup‐shape membrane‐limiting vesicles with an average 67.4 ± 8.6 nm minimum Feret's diameter in the purified material (*Figure* 2C). NTA showed a population of vesicles in a concentration of 2.9 10^10^ ± 2.6 10^9^ particles/mL and 75.2 ± 7.5 nm in size (*Figure* 2D), which is in agreement with the particle size distribution resulted from electron microscopy analysis. Based on these results we can classify our vesicle population as sEVs (hereon sEVs) subtype. Through western blot analysis, tetraspanins CD81 and CD9, ALIX and the heat shock protein HSC70, some of the EV‐associated markers recommended by MISEV2023 guidelines,^9^ were identified to be enriched while very low levels of non‐vesicular protein CALNEXIN were confirmed in our samples (*Figure* 2E). The same amount of total protein was loaded in all gels. These data confirm the presence and purity of EV in our plasma isolates and, therefore, the suitability of our method. Moreover, the list of 157 proteins identified by proteomic analysis in both sarcopenic and robust samples (*Table* S1) was compared with datasets from two online open access repositories of experimental data from studies on EVs, ExoCarta (Version 5; 29 July 2015) [S8] and Vesiclepedia (Version 4.1; 15 August 2018) [S9]. A Venn diagram was constructed based on the combinations of those three lists. Results showed a large overlap between the proteins identified in the proteomic analysis and those reported in ExoCarta and Vesiclepedia when compared with each other (*Figure* 2F, *Table* S2). Specifically, 109 (69%) of the 157 proteins identified were found in, at least, one of the two databases, 97 (62%) in the ExoCarta database, 39 (25%) in the Vesiclepedia database and 27 (17%) in both of them. These data confirmed that the sEVs in our preparations contained previously reported EV proteins. Interestingly, 48 EV proteins were found de novo in our samples, which have not been reported in the ExoCarta nor Vesiclepedia databases. To our knowledge, this is the first time that these proteins have been described in association with EVs and in particular with small EVs.

### Proteomic analysis of plasma sEVs reveals differentially contained proteins in sarcopenic patients

**Figure 3:**
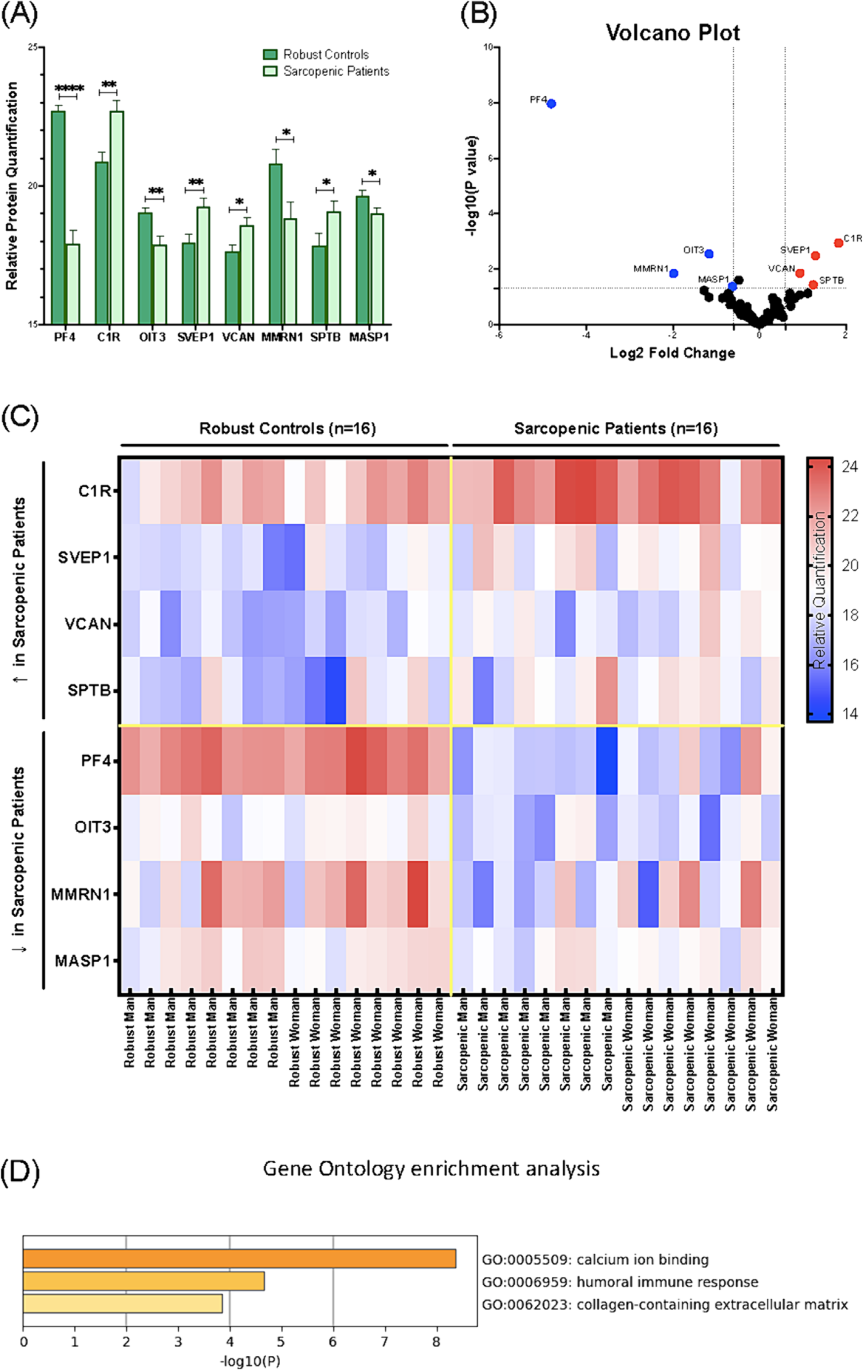
Proteomics analysis of plasma small extracellular vesicles (sEVs) in sarcopenic patients and robust controls for the discovery cohort. (*A*) Bar graph showing the comparison in the relative quantification of candidate proteins between groups. Data represent the mean value ± standard error of the mean (*SEM*). (*B*) Volcano plot of differentially concentrated proteins. The data are shown as *x* (Log2FC) − *y* (Log10 *P* value) scatter plots. Blue dots represent depleted proteins; red dots represent enriched proteins. (*C*) Heatmap of differentially expressed proteins. (*D*) Bar graph of the top‐level gene ontology biological processes across candidate proteins, coloured by *P* values via Metascape. The enriched terms in the biological process, cellular component, and molecular functions were listed. For comparisons between the two groups of the discovery cohort (*n* = 16 for sarcopenic patients; *n* = 16 for robust control), two‐tailed Student's *t* test was used. *P < 0.05, **P < 0.01, ***P < 0.001, and ****P < 0.0001.

Among the 157 proteins identified in the present study, 8 proteins were differentially contained in sEVs obtained from sarcopenic vs robust individuals. Four of these eight proteins were lower in sarcopenic patients (PF4, OIT3, MMRN1 and MASP1) and four showed higher concentration (C1R, SVEP1, VCAN and SPTB) (*Table* 2). Hereon, we defined them as our candidate proteins. The downregulated proteins in sarcopenic patients were platelet factor 4, also known as CXCL4 (PF4, *P* = 2.3E‐10; log2 FC = 4.806), oncoprotein‐induced transcript 3 protein (OIT3, *P* = 0.002401; log2 FC = −1.161), Multimerin‐1 (MMRN1, *P* = 0.014449; log2 FC = −1.982) and Mannan‐binding lectin serine protease 1 (MASP1, *P* = 0.042872; log2 FC = ‐0.6268) (*Figure* 3A–C). In order of magnitude, PF4 showed the strongest reduction in sarcopenic patients (21.14% compared with the robust group). followed by MMRN1 (9.56%), OIT3 (6.09%) and MASP1 (3.21%). On the other hand, the upregulated proteins in sarcopenic patients were Complement C1r subcomponent (C1R, *P* = 0.001144; log2 FC = 1.83), Sushi von Willebrand factor type A, EGF and pentraxin domain‐containing protein 1 (SVEP1, *P* = 0.003339; log2 FC = 1.295), versican core protein (VCAN, *P* = 0.013988; log2 FC = 0.9373), spectrin beta chain and erythrocytic (SPTB, *P* = 0.03672; log2 FC = 1.243). The results show an 8.77% higher C1R protein concentration in sarcopenic patients sEVs with respect to the robust group, closely followed by SVEP (7.24%), SPTB (7%) and VCAN (5.33%). More information is detailed in *Table* S3. The functional enrichment analysis of the differentially contained proteins indicated an enrichment in pathways related to the calcium ion binding, the collagen‐containing extracellular matrix (ECM) and the immune system process, specifically the humoral immune response (*P* < 0.05) (*Figure* 3D). Protein–Protein Interaction Networks Functional Enrichment Analysis of candidate proteins and predicted functional partners showed a dense interaction between all the candidate proteins, except for protein SPTB (*Figure* S1A). The top‐level GO molecular functions across candidate proteins was calcium ion binding (*Figure* S1B) while the most enriched GO mechanism was humoral immune system (*Figure* S1C).

**Table 2:** List of differentially contained proteins in plasma sEVs from sarcopenia patients as compared with robust individuals.

Uniprot	Gene name	Protein names	Mean of sarcopenic patients	Mean of robust controls	*P value*		Difference (log2 fold change)
P02776	PF4	Platelet factor 4 (PF‐4) (C‐X‐C motif chemokine 4) (Iroplact) (Oncostatin‐A) [Cleaved into: Platelet factor 4, short form (Endothelial cell growth inhibitor)]	17.91	22.71	2.30E‐10	****	−4.806
P00736	C1R	Complement C1r subcomponent (EC 3.4.21.41) (Complement component 1 subcomponent r) [Cleaved into: Complement C1r subcomponent heavy chain; Complement C1r subcomponent light chain]	22.69	20.86	0.0011	**	1.83
Q8WWZ8	OIT3	Oncoprotein‐induced transcript 3 protein (Liver‐specific zona pellucida domain‐containing protein)	17.88	19.04	0.0024	**	−1.161
Q4LDE5	SVEP1	Sushi, von Willebrand factor type A, EGF and pentraxin domain‐containing protein 1 (CCP module‐containing protein 22) (Polydom) (Selectin‐like osteoblast‐derived protein) (SEL‐OB) (Serologically defined breast cancer antigen NY‐BR‐38)	19.26	17.96	0.0033	**	1.295
P13611‐2	VCAN	Versican core protein (Chondroitin sulfate proteoglycan core protein 2) (Chondroitin sulfate proteoglycan 2) (Glial hyaluronate‐binding protein) (GHAP) (Large fibroblast proteoglycan) (PG‐M)	18.57	17.63	0.014	*	0.937
Q13201	MMRN1	Multimerin‐1 (EMILIN‐4) (Elastin microfibril interface located protein 4) (Elastin microfibril interfacer 4) (Endothelial cell multimerin) [Cleaved into: Platelet glycoprotein Ia; 155 kDa platelet multimerin (p‐155) (p155)]	18.83	20.82	0.0144	*	−1.982
P11277‐2	SPTB	Spectrin beta chain, erythrocytic (Beta‐I spectrin)	19.1	17.85	0.0367	*	1.243
P48740‐2	MASP1	Mannan‐binding lectin serine protease 1 (EC 3.4.21.‐) (Complement factor MASP‐3) (Complement‐activating component of Ra‐reactive factor) (Mannose‐binding lectin‐associated serine protease 1) (MASP‐1) (Mannose‐binding protein‐associated serine protease) (Ra‐reactive factor serine protease p100) (RaRF) (Serine protease 5) [Cleaved into: Mannan‐binding lectin serine protease 1 heavy chain; Mannan‐binding lectin serine protease 1 light chain]	19.01	19.64	0.0429	*	−0.627

*Note*: Student's *t*‐tests were performed among the proteins identified by mass spectrometry in both sarcopenic and robust samples in order to compare their concentrations between the groups and determine possible deregulated proteins. *P* value of <0.05 was considered statistically significant.

*P < 0.05, **P < 0.01, ***P < 0.001, and ****P < 0.0001.

In order to study whether the concentration of these proteins was influenced or not by the sex of the participants, two‐way ANOVA test followed by Bonferroni post‐hoc were performed. Our results showed no gender‐related differences in any of the proteins significantly contained in the sarcopenia sEVs vs. robust controls (*Figure* S2 and *Table* S4). Moreover, aware of the differences in age means between robust and sarcopenic group, we confirmed by ANOVA analysis that age was not underlying the significant differences in PF4 and C1R concentrations between patients and controls (*Figure* S3 and *Table* S5).

### The levels of the candidate proteins correlate with sarcopenia diagnostic tests scores

With the aim of evaluating the diagnostic power of the eight candidate proteins, their concentrations were correlated with the EWGSOP2 sarcopenia tests for low strength and low physical performance, specifically the *Grip strength, Chair stand, SMI, SPPB, Gait speed* and *TUG 3 m tests* (*Table* 3) in the discovery cohort. Six out of the eight candidate proteins significantly correlated with two or more diagnostic tests while MMRN1 and MASP1 showed no correlation with any of the physical tests performed. *Chair stand* test showed significant correlation in six out of the eight proteins analysed, followed by the *TUG 3 m, SSPB* and *Gait speed* tests in which correlated with five out of the eight proteins. PF4, C1R and SPTB concentrations significantly correlated with all four tests performed, being PF4 and C1R the strongest correlations (*Figure* S4).

**Table 3:** Correlation of the scores for the recommended sarcopenia diagnostic tests and the candidate proteins.

	PF4	C1R	SPTB	SVEP1	OIT3	VCAN	MMRN1	MASP1
Chair stand (s)	−0.735****	0.567***	0.409*	0.458**	−0.406*	0.361*	−0.217	−0.173
TUG 3 m (s)	−0.730****	0.486**	0.385*	0.441*	−0.387*	0.327	−0.32	−0.237
SPPB (points)	0.7156****	−0.619***	−0.370*	−0.499**	0.436*	−0.246	0.285	0.196
Gait speed (m/s)	0.684****	−0.648****	−0.398*	−0.515**	0.383*	−0.316	0.176	0.117
Grip strength (kg)	0.430*	−0.434*	−0.402*	−0.337	0.32	−0.426*	0.005	0.137

*Note*: Data indicate Spearman–rho correlation coefficients in the discovery cohort (*n* = 16 for sarcopenic patients; *n* = 16 for robust control).

Abbreviations: SPPB, Short Physical Performance Battery; TUG, Time Up and Go 3 m.

*P < 0.05, **P < 0.01, ***P < 0.001, and ****P < 0.0001.

Within PF4*, Chair stand test* showed the strongest correlation (*r* = −0.735, *P =* 2.4E‐06), closely followed by *TUG 3 m* (*r* = −0.730, *P =* 3.2E‐06), being negative this correlation for both tests. On the other hand, PF4 positively correlated with *Gait Speed* (*r* = 0.684, *P* = 1.6E‐05) and with *Grip Strength* (*r* = 0.430, *P =* 0.0157). In the case of C1R, the strongest correlation was found with *Gait Speed* test (*r* = −0.722, *P =* 4.6E‐05), followed by the *SPPB* (*r* = −0.7040, *P* = 9.9E‐05) and *Chair Stand* (*r* = 0.705, *P* = 1.4E‐05). Finally, C1R also correlates with *TUG 3 m* (*r* = 0.619, *P =* 0.0003) and *Grip Strength* (*r* = −0.559, *P =* 0.0013).

### Validation of sEV PF4 and C1R as diagnostic read outs for sarcopenia

**Figure 4:**
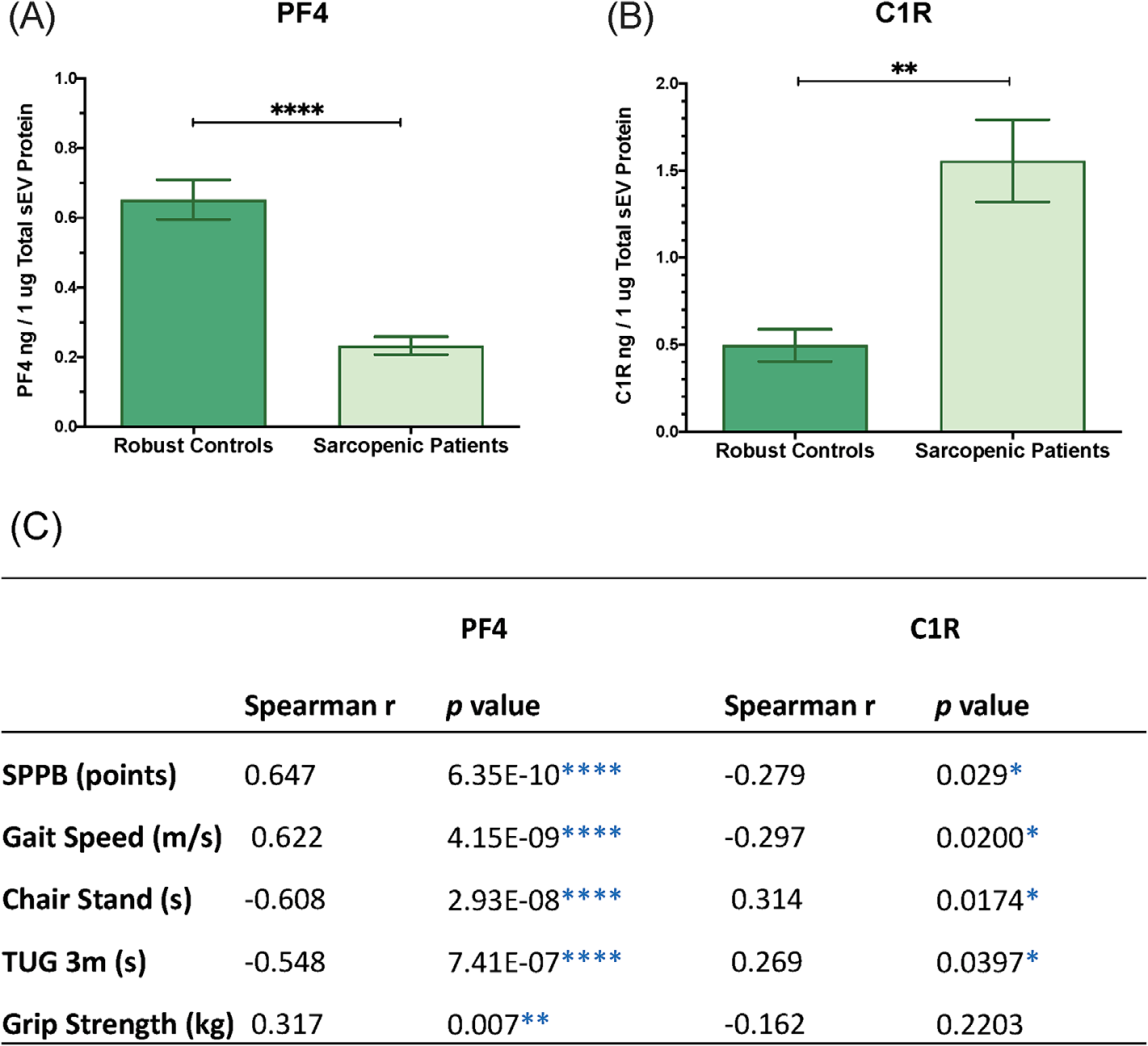
Validation of PF4 (*A*) and C1R (*B*) levels in plasma small extracellular vesicles (sEVs) from robust controls and sarcopenic patients. ELISA quantification was performed on the validation cohort (*n* = 49 for sarcopenic patients; *n* = 29 for robust control). Protein levels were normalized to the total sEV protein content. Data represent the mean value ± standard error of the mean (SEM), and statistical analysis was performed using Mann–Whitney test. (*C*) Correlation between the scores of the main diagnostic tests and the concentration of PF4 and C1R. TUG, time up and go 3 m. *P < 0.05, **P < 0.01, ***P < 0.001, and ****P < 0.0001.

Among the candidate proteins, PF4 and C1R showed the largest differences in concentration within sEVs between the sarcopenia and robust individuals. Therefore, these proteins were chosen for validation as candidate diagnosis biomarkers for the disease. With this aim C1R and PF4 were quantified by ELISA in sEVs obtained from *the validation cohort*, consisting of 49 sarcopenic patients and 29 robust controls. ELISA analysis showed a lower concentration of PF4 (*P* = 1.1E‐09) and C1R elevated (*P* = 0.001287) in sarcopenic patients (*Figure* 4A,B), further confirming the mass spectrometry results. Specifically, for PF4 the mean was 0.2324 ng/μg total sEV protein (*SEM* ± 0.026) and 0.6522 ng/μg total sEV protein (*SEM* ± 0.057) in the sarcopenic and robust samples, respectively, which represents a 64.37% reduction in this protein in sarcopenic group. For C1R, the mean was 1.556 ng/μg total sEV protein (*SEM* ± 0.236) and 0.4957 ng/μg total sEV protein (*SEM* ± 0.093) in the sarcopenic and robust samples respectively, which represents an increase of 213.90% with respect to the robust group. More information of this analysis is available in *Table* S6. Again, no significant differences were found between men and women samples when performing ANOVA analysis, confirming the findings of the proteomic analysis from the discovery cohort (*Figure* S5 and *Table* S7). To test whether the difference in PF4 concentration between the two groups was EV‐specific, ELISA tests were performed on whole plasma samples and on the rest of the size exclusion chromatography non‐EV fractions. No significant differences were found in the amount of PF4/total protein in any of the cases (*Figure* S6).

When calculated the correlations between the concentration of the selected proteins and the main physical tests recommended by EWGSOP2 for the diagnosis of sarcopenia.^4^ For PF4, all correlations were statistically significant and, in the case of the *Grip Strength test*, significance even improved from *P =* 0.014 to *P =* 0.007. For C1R, all correlations, except for *Grip Strength test*, were statistically significant (*Figure* 4C). Hence, the significant difference and correlation with diagnostic tests remained in the larger validation cohort which confirmed that both proteins were altered in the disease and are robust candidates to be diagnostic biomarkers of sarcopenia. Muscle mass was only evaluated in frail patients showing low muscle strength to confirm sarcopenia, following EWGSOP2 algorithm (*Figure* S7). Correlation between PF4 and C1R and muscle mass tests, specifically ASM and SMI, in frail patients revealed no statistically association, suggesting that these proteins might be more closely related to muscle strength and function rather than muscle mass. In fact, several studies have shown uncoupling of muscle mass‐strength‐function which is particularly evident in older persons [S11, S12].

**Figure 5:**
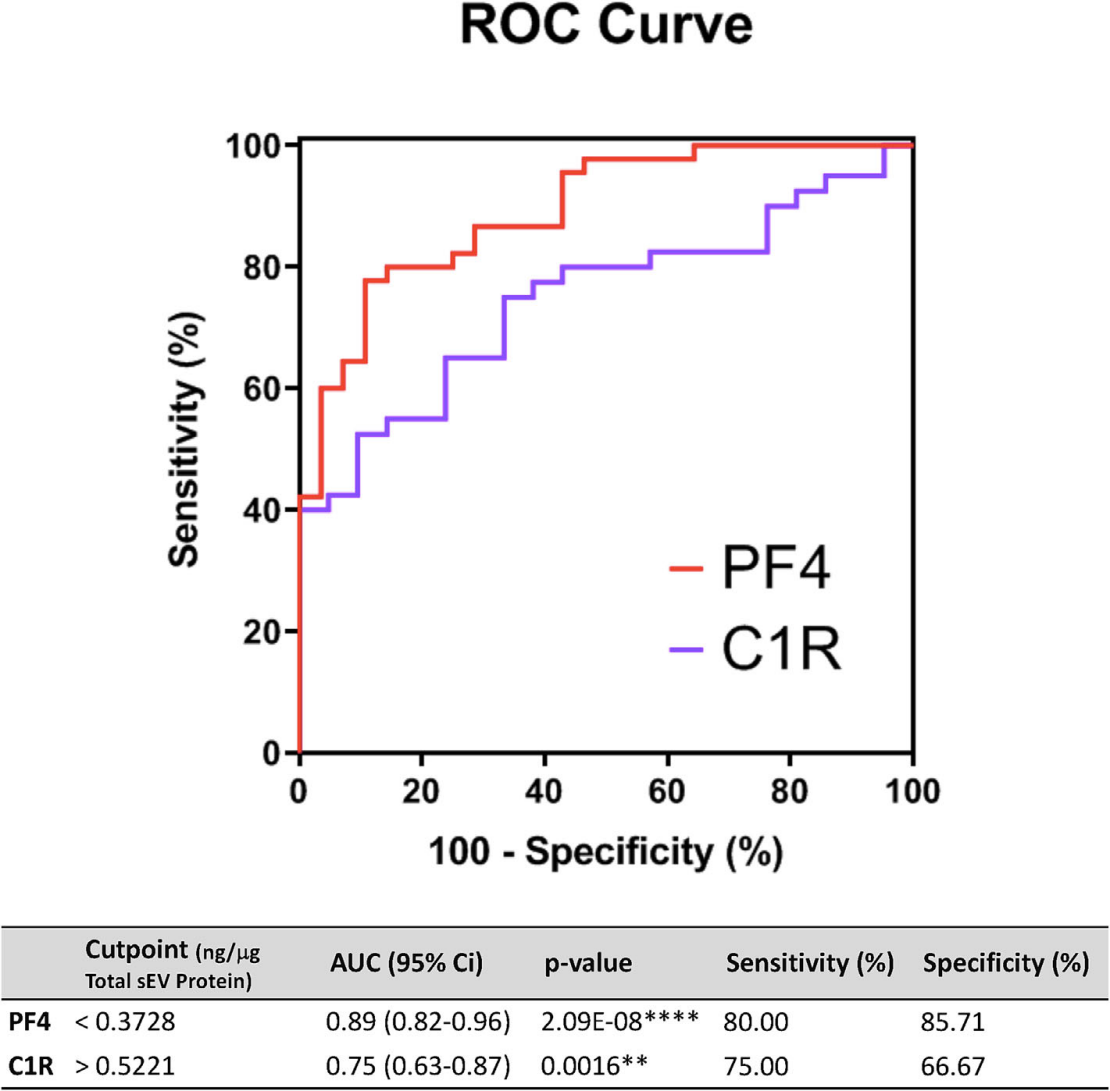
ROC curve of PF4 and C1R for the diagnosis of sarcopenia. The optimal cut‐off values for PF4 and C1R with their respective sensitivity and specificity measures, are shown in the table. AUC, area under the curve; ROC, receiver operating characteristic. *P < 0.05, **P < 0.01, ***P < 0.001, and ****P < 0.0001.

To evaluate the diagnostic power of PF4 and C1R in sarcopenia, a ROC curve analysis was performed with the data obtained from the validation set. AUC values can range from 0 to 1, with 0 indicating a test with zero diagnostic power and 1 indicating perfect diagnostic power in terms of sensitivity and specificity. In general, AUC values of 0.5 are considered poor diagnostics; 0.7–0.8 is considered acceptable; 0.8 to 0.9 is considered excellent and more than 0.9 is considered exceptional [S10]. As shown in the table below, PF4 protein has an AUC value of 0.89 [95% confidence interval (CI): 0.82–0.96], so its diagnostic power for sarcopenia would be considered excellent to exceptional. For C1R, AUC was 0.75 (95% CI: 0.63–0.87) (*Figure* 5). The optimal cut‐off value for PF4, which maximize both sensitivity and specificity was 0.3728 ng PF4/μg total sEV protein corresponding that threshold with an 80% sensitivity and 85.71% specificity when diagnosing a patient as sarcopenic. In the case of C1R, a concentration above 0.5221 ng C1R/μg total sEV protein would allow sarcopenia diagnosis with 75% sensitivity and 66.67% specificity.

## Discussion

Sarcopenia, a decline in skeletal muscle strength, mass and function with ageing, is a paramount geriatric issue owing to its clinical and socioeconomic implications. The ageing demography exacerbates its global economic impact on healthcare, accounting for direct and indirect costs.^2^, ^21^ To date, identification of sarcopenia and frailty has been based on clinical, functional and imaging parameters,^3^, ^4^, ^5^, ^6^, ^7^ which all need specific equipment and skilled operators, unavailable at a regular basis in primary care centres and nursing homes or not applicable to all patients. As a result, there is a current underdiagnosis and undertreatment of the disease.^8^ It is therefore necessary to develop novel disease diagnostic instruments that are sensitive, specific and easy to perform and interpret and do not require costly and highly specialized personnel or infrastructure. As an alternative to these traditional diagnostic methods and given the multifactorial nature of sarcopenia (involving neuromuscular, endocrine and protein functional alterations^1^), evaluation of a panel composed of multiple biological circulating or tissue biomarkers may fill this gap. However, the lack of a univocal definition for sarcopenia and its underlying complex pathophysiology represents a burden.

In the era of ‘‐omics’ sciences, many researchers have used these methods to identify reliable circulating or tissue biomarkers. Particularly, within the field of proteomics, research has gained attention on the protein content of EVs. Recent studies indicate that EVs play an important role in many aspects of ageing such as telomere shortening, apoptosis, autophagy or mitochondrial damage and are important mediators of the effects of senescent cells on their microenvironment.^22^ In this line, in 2020 Basisty *et al*. characterized the composition of plasma EVs released by senescent cells identifying several biomarkers of cellular senescence in human plasma, including growth/differentiation factor 15 (GDF15), stanniocalcin 1 (STC1) and serine protease inhibitors (SERPINs).^23^ A growing body of literature highlights the role of EVs for skeletal muscle physiology and wasting.^14^, ^15^, ^16^ In this line, Shao *et al*. showed in 2022 that, during the ageing process, EV miR‐690 derived from atrophic skeletal muscle fibres, inhibited the differentiation of satellite cells.^23^ However, little is known on the potential use of specific EV cargo to diagnose muscle‐related disorders such as sarcopenia.

In this study, we have profiled for the first time the proteomic signature of sarcopenia human plasma sEVs. 157 EV proteins were reported, being 48 of them previously unreported within ExoCarta and Vesiclepedia databases. Eight of these proteins presented different concentrations in sarcopenic patients as compared with robust individuals (PF4, OIT3, MMRN1, MASP1, C1R, SVEP1, VCAN and SPTB) and could be used as diagnostic biomarker in the disease. Indeed, the concentration of most of the candidate proteins, and particularly that of PF4 and C1R correlated with the scores of the tests recommended by EWGSOP2 to quantify muscle strength and function. These results have been validated in two cohorts of patients and with two different laboratory techniques, proving their robustness and reliability. On the other hand, our analysis demonstrated that these proteins are not sex‐specific, which reinforces their utility as a diagnostic biomarker both for men and women.

Looking at the biological function of these proteins in the organism, the top one enriched pathway is the calcium ion binding including six out of the eight candidate proteins differentially contained in patient samples (OIT3, MMRN1, VCAN, C1R, MASP1 and SVEP1). Calcium ions play a pivotal role in skeletal muscle function, plasticity and disease. They serve as the primary regulatory and signalling molecules in all muscle types and are intricately connected with the control of contraction and relaxation.^24^ Interestingly, a significant reduction of the amount of available Calcium in the sarcoplasmic reticulum of aged skeletal muscle has been observed.^25^ In fact, several studies have highlighted alterations in various calcium signalling and handling molecules such as calsequestrin‐1, sarcalumenin, myozenin‐1, annexins or dystrophin in ageing and muscle diseases for example, Duchenne muscular dystrophy.^26^ These data are in line with our results and reveal a previously unreported role of sEVs in calcium metabolism and thus muscle function. Immune response and inflammation are also highly represented in the GO analysis of the sEV proteomics. Extensive bibliography exists on the correlation between ageing and systemic levels of inflammatory molecules,^27^ being this chronic low‐grade inflammation state a potential trigger for age‐related diseases such as sarcopenia.^28^ Our findings are consistent with these studies and suggest that proteins contained in sarcopenia plasma sEVs reflect an exacerbated inflammatory state associated with the disease. A very recent in vivo study using mouse muscles showed an increase in a macrophage subpopulation, together with collagen accumulation during ageing, which were suggested to contribute to functional decline in aged skeletal muscle. These authors proposed that macrophages in aged muscle might be regulating skeletal muscle ECM remodelling through collagen deposition and, thus, underlying scarring.^29^ Collagen is the main structural protein in the skeletal muscle ECM, which is widely known to be essential for the mechanical support of muscles and maintenance of tissue integrity.^30^ In this line, further studies have demonstrated an increase in collagen content and alignment within ECM in aged muscle which result in an increase in skeletal muscle stiffness and rigidity.^31^

Platelet factor 4 (PF4) is a protein member of the CXC chemokine family, also called CXCL4. This chemokine is released from the alpha granules of activated platelets and has high affinity for heparin being involved in platelet aggregation.^32^ Although these authors cannot rule out the possibility that traces of proteins from platelet granules are co‐eluded with sEVs, strong and multiple EV markers have been detected in the samples subjected to proteomic and ELISA analyses. Moreover, both sarcopenia and robust patient samples were processed following the same protocol; thus, potential technical limitations are not likely to underlie major differences observed in PF4 and C1R levels.

Other studies report a correlation between PF4 and the vasculature, playing a pro‐atherogenic role while also having anti‐angiogenic effects inhibiting endothelial cell growth.^32^ A very recent study observed that circulating levels of PF4 were decreased in blood plasma preparations of old mice and humans relative to younger individuals and the systemic administration of exogenous PF4 improved cognition and attenuated neuroinflammation in aged mice, which would support a pro‐cognition and anti‐inflammatory role for this protein.^33^ The number of studies that relate this protein to skeletal muscle are scarce. A 2023 review concluded that platelet‐derived factors play a vital role in tissue regeneration, particularly in skeletal muscle in which chemokines recruit neutrophils to muscles after injury. Disrupting this early step hampers muscle regeneration, leading to increased inflammation, compromised neo‐angiogenesis, reduced myofiber growth and diminished post‐injury muscle force.^34^ These findings would be in line with our results, as we observed a decrease in PF4 levels in patients with an altered muscle strength, mass and function.

Complement C1r subcomponent (C1R) is a serine protease that heterotrimerize with C1Q and C1S to form C1, the first component of the classical pathway of the complement system. The complement system acts as a mediator in the innate immune response by ultimately triggering phagocytosis and inflammation, and intervening in processes as relevant to the cell as mitochondrial function and Wnt signalling pathways.^35^ The regulation and magnitude of complement responses can influence ageing‐related changes across different tissues including skeletal muscle.^36^ This was demonstrated by Naito et al. who determined that the rise in C1Q secretion induced by ageing triggers the activation of the Wnt signalling pathway within muscles, leading to muscle fibrosis.^37^ These results were later supported by Watanabe *et al*. whose analysis revealed that serum C1Q levels exhibited an upward trend with advancing age, and these higher levels were negatively correlated with both muscle mass and strength. Moreover, it was observed that a 12 week resistance training intervention in older adults effectively mitigated the age‐related increase in serum C1Q levels.^38^ Most recently, both C1R and C1S subunits contribution to the development of Duchenne muscular dystrophy (DMD) by regulating innate immunity and inflammation has been studied.^39^ In fact, their pharmacological inhibition in a murine model of DMD mitigated the activation of the WNT signalling pathway, reduced the fibrosis and ameliorated the dystrophic phenotype.^40^ Considering our results, it will be interesting to further investigate the role of the complement system and other immunological molecules in sarcopenia.

In this study, we report for the first time an association between EV plasma PF4 and C1R level with age‐related loss of muscle strength in humans while the specific mechanisms involved remains nuclear. This novel study opens the door to the use of EV proteins as a diagnostic method for sarcopenia, although further investigation of the specific mechanisms and roles of the molecules identified in the disease are still needed.

## Conclusions

Here we present the first proteomic analysis of human circulating EVs in sarcopenia. Our data suggest that circulating EVs from sarcopenic patients carry specific proteins related to calcium and collagen metabolism, immune response and inflammation, which will deserve further exploration in the pathophysiology of the disease.

Moreover, PF4 and C1R proteins arise as promising diagnostic biomarkers for the identification of sarcopenia in routine clinical practice facilitating prevention and early and more effective interventions.

## Conflict of Interest

P. A., D. N. V., T. L. R., R. O., R. M. and G. V. R declare that a patent application with number EP24382593 has been submitted regarding the work included in this manuscript.

## Funding information

This research was supported by the *European Co* *mmission* Horizon 2020 framework Programme Marie Curie individual fellowship scheme (Fellowship 752349), *University of Zaragoza* Young investigator call (JIUZ‐2021‐BIO‐05) and *Gobierno de España:* *Ministerio de Ciencia e Innovación* (PID2022‐140911OB‐I00) granted to R. M. *Gobierno de España:* *Ministerio de Economía, Industria y Competitividad* (DEP2016‐78309‐R), *University of Zaragoza* (UZ2021‐BIO‐05), *Centro Universitario de la Defensa de Zaragoza* (UZCUD2017‐BIO‐01), *Con* *sorcio Centro de Investigación Biomédica en* *Red* *Fragilidad y Envejecimiento Saludable* (CIBERFES, CB16/10/00477 and CB12/03/30038) granted to G. V. R. *Instituto de Salud Carlos III and Fondo Europeo de Desarrollo Regional* (FEDER) ‘Una manera de hacer Europa’ (PI21/00372), *Centro de Investigación Biomédica en Red sobre Enfermedades Neurodegenerativas* (CIBERNED, CB18/05/0037) granted to R. O. P. A. was supported by the *Departamento de Industria e Innovación from Gobierno de Aragón and Fondo Social Europeo*. V. S. acknowledges the funding from *Fundación Ramón Areces* (XX concurso nacional‐ciencias de la vida y la materia) and the access to ELECMI‐LMA ICTS.

## Supporting information


**Figure S1** **Enrichment analyses of candidate proteins.** (A) Protein–Protein Interaction Networks Functional Enrichment Analysis of candidate proteins and predicted functional partners by STRING. (B) Bar graph of the top‐level Gene Ontology Molecular Functions across candidate proteins, coloured by p‐values via Metascape. (C) Bar graph of the top‐level Gene Ontology mechanisms enriched across candidate proteins, coloured by p‐values via Metascape.

**Figure S2** **Analysis of proteomics results of candidate proteins quantification in plasma sEVs between sarcopenic patients and robust controls of the discovery cohort segregated by sex.** Two‐way univariate analysis of variance (ANOVA) and Bonferroni post‐hoc tests were performed to identify the effect of the disease (sarcopenic/robust) and sex (men/women) in the concentrations of the candidate proteins (*n* = 16 for Sarcopenic Patients; n = 16 for Robust Control, 8 men and 8 women in each group). Data represent the mean value ± standard error of the mean (SEM).

**Figure S3** **Validation of PF4 and C1R levels in plasma sEVs from robust controls and sarcopenic patients stratified by age.** ELISA quantification was performed on the validation cohort (*n* = 49 for sarcopenic patients; *n* = 29 for robust control). Protein levels were normalized to the total sEV protein content. Data represent the mean value ± standard error of the mean (SEM), and statistical analysis was performed using two‐way univariate analysis of variance (ANOVA) and Bonferroni post‐hoc tests test. Bar graph of the difference in PF4 (A) and C1R (B) plasma concentration between sarcopenic patients and robust controls stratified by age.

**Figure S4** **Correlation of the scores of the main diagnostic tests for sarcopenia and the levels of sEV PF4 (A) and C1R (B) in the discovery cohort.** Data indicate Spearman‐rho correlation coefficients. *n* = 16 for sarcopenic patients; n = 16 for robust control. TUG = Time Up and Go 3 meters; SPPB = Short Physical Performance Battery.

**Figure S5** **Validation of PF4 and C1R levels in plasma exosomes from robust controls and sarcopenic patients stratified by sex.** ELISA quantification was performed on the validation cohort (*n* = 49 for sarcopenic patients; *n* = 29 for robust control). Protein levels were normalized to the total sEV protein content. Data represent the mean value ± standard error of the mean (SEM), and statistical analysis was performed using two‐way univariate analysis of variance (ANOVA) and Bonferroni post‐hoc tests test. Bar graph of the difference in PF4 (A) and C1R (B) plasma concentration between sarcopenic patients and robust controls segregated by sex.

**Figure S6** **Comparison of PF4 levels in different types of samples.** (A) ELISA quantification on representative plasma, SEC Non‐EV samples and SEC EV‐enriched samples from sarcopenic patients and robust controls. Protein levels were normalized to the total protein content of each sample. Data represent the mean value ± standard error of the mean (SEM), and statistical analysis was performed using two‐tailed Student's t‐test.

**Figure S7.** **Correlation of the scores of the main low muscle mass diagnostic tests for sarcopenia and the levels of sEV PF4 (A,C) and C1R (B,D) in sarcopenic patients of discovery (A,B) and validation (C,D) cohort**. Data indicate Spearman‐rho correlation coefficients. *n* = 16 for sarcopenic patients in discovery cohort; *n* = 49 for sarcopenic patients in validation cohort. ASM = Appendicular Skeletal Muscle; SMI = Skeletal Muscle Mass Index.

**Data S1.** Supporting Information.

**Table S1** Proteins identified by proteomic analysis of plasma sEVs from sarcopenia patients as compared to robust individuals. 157 proteins were identified by mass spectrometry in both sarcopenic and robust samples of the discovery cohort (*n* = 16 for Sarcopenic Patients; n = 16 for Robust Control, 8 men and 8 women in each group).
Table S2 List of overlapped proteins between the proteins identified in the proteomic analysis and those reported in ExoCarta and Vesiclepedia when compared with each other. ExoCarta (Version 5; 29 July 2015) and Vesiclepedia (Version 4.1; August 15, 2018).
Table S3 List of differentially contained proteins in plasma sEVs from sarcopenia patients as compared to robust individuals. Student's t‐tests were performed among the proteins identified by mass spectrometry in both sarcopenic and robust samples of the discovery cohort (*n* = 16 for Sarcopenic Patients; n = 16 for Robust Control) in order to compare their concentrations between the groups and determine possible deregulated proteins. *P* value of <0.05 was considered statistically significant.
Table S4 Analysis of proteomics results of candidate proteins quantification in plasma sEVs between sarcopenic patients and robust controls of the discovery cohort segregated by sex. Two‐way univariate analysis of variance (ANOVA) and Bonferroni post‐hoc tests were performed to identify the effect of the disease (sarcopenic/robust) and sex (men/women) in the concentrations of the candidate proteins (*n* = 16 for Sarcopenic Patients; n = 16 for Robust Control, 8 men and 8 women in each group). *P* value of <0.05 was considered statistically significant.
Table S5 Validation of PF4 and C1R levels in plasma sEVs from robust controls and sarcopenic patients stratified by age. ELISA quantification was performed on the discovery cohort (*n* = 16 for Sarcopenic Patients; n = 16 for Robust Control, 8 men and 8 women in each group). Protein levels were normalized to the total sEV protein content and statistical analysis was performed using two‐way univariate analysis of variance (ANOVA) and Bonferroni post‐hoc tests test. *P* value of <0.05 was considered statistically significant.
Table S6 Validation of PF4 and C1R levels in plasma sEVs from robust controls and sarcopenic patients. ELISA quantification was performed on the validation cohort (*n* = 49 for sarcopenic patients; *n* = 29 for robust control). Protein levels were normalized to the total sEV protein content and statistical analysis was performed using Mann–Whitney test. *P* value of <0.05 was considered statistically significant.
Table S7 Validation of PF4 and C1R levels in plasma sEVs from robust controls and sarcopenic patients stratified by sex. ELISA quantification was performed on the validation cohort (*n* = 49 for sarcopenic patients; *n* = 29 for robust control). Protein levels were normalized to the total sEV protein content and statistical analysis was performed using two‐way univariate analysis of variance (ANOVA) and Bonferroni post‐hoc tests test. *P* value of <0.05 was considered statistically significant.

**Data S2.** Supporting Information.
