# Supplementary figures and images for: Proteomic profiling of human plasma extracellular vesicles identifies PF4 and C1R as novel biomarker in sarcopenia
Source: J Cachexia Sarcopenia Muscle. 2024 Jul 15;15(5):1883–97. doi: 10.1002/jcsm.13539 (PMC11446689; doi:10.1002/jcsm.13539)

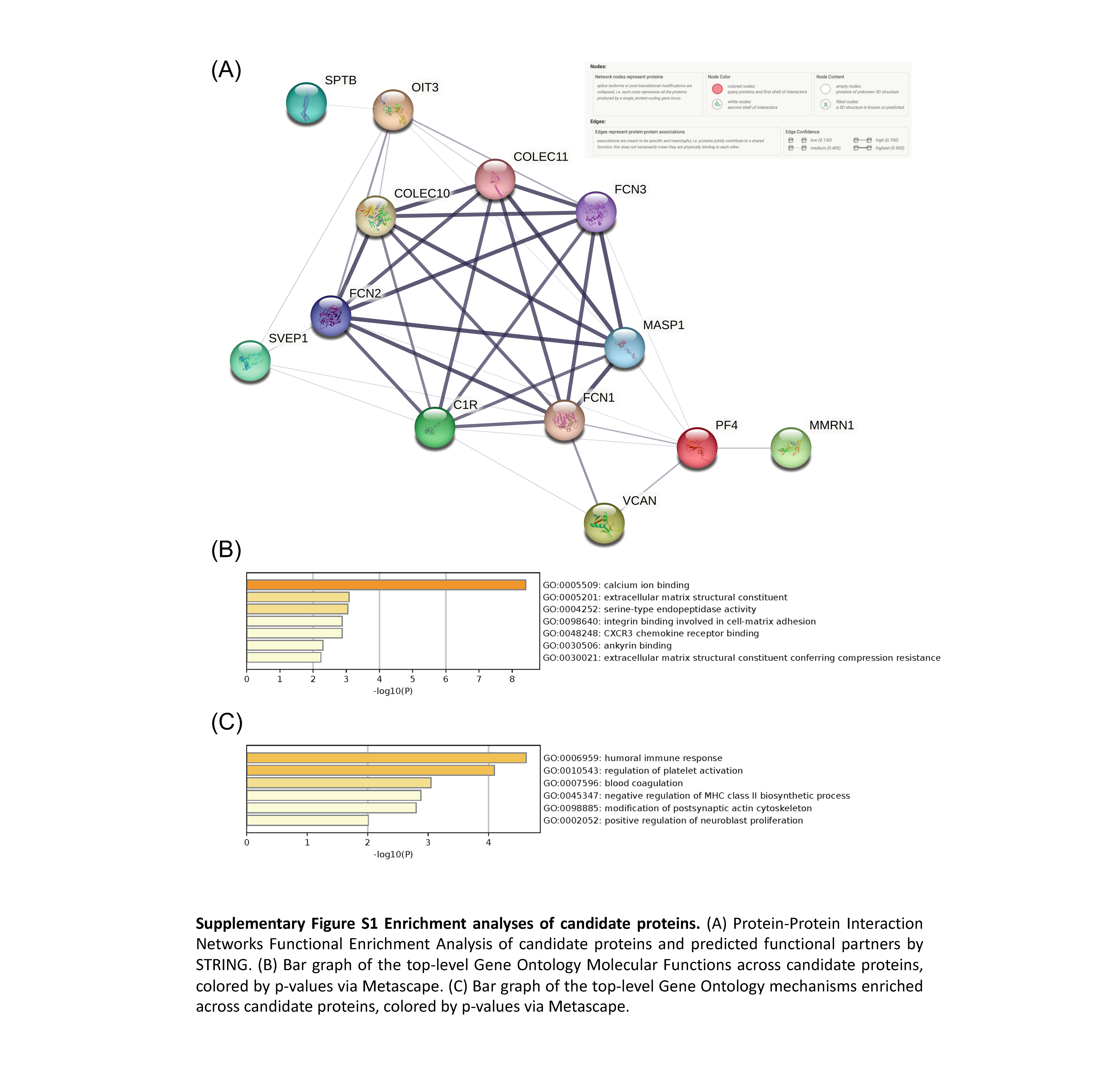

Supplement: Supplementary file 1 — Figure S1 Enrichment analyses of candidate proteins. (A) Protein–Protein Interaction Networks Functional Enrichment Analysis of candidate proteins and predicted functional partners by STRING. (B) Bar graph of the top‐level Gene Ontology Molecular Functions across candidate proteins, coloured by p‐values via Metascape. (C) Bar graph of the top‐level Gene Ontology mechanisms enriched across candidate proteins, coloured by p‐values via Metascape. [file JCSM-15-1883-s008.tiff]

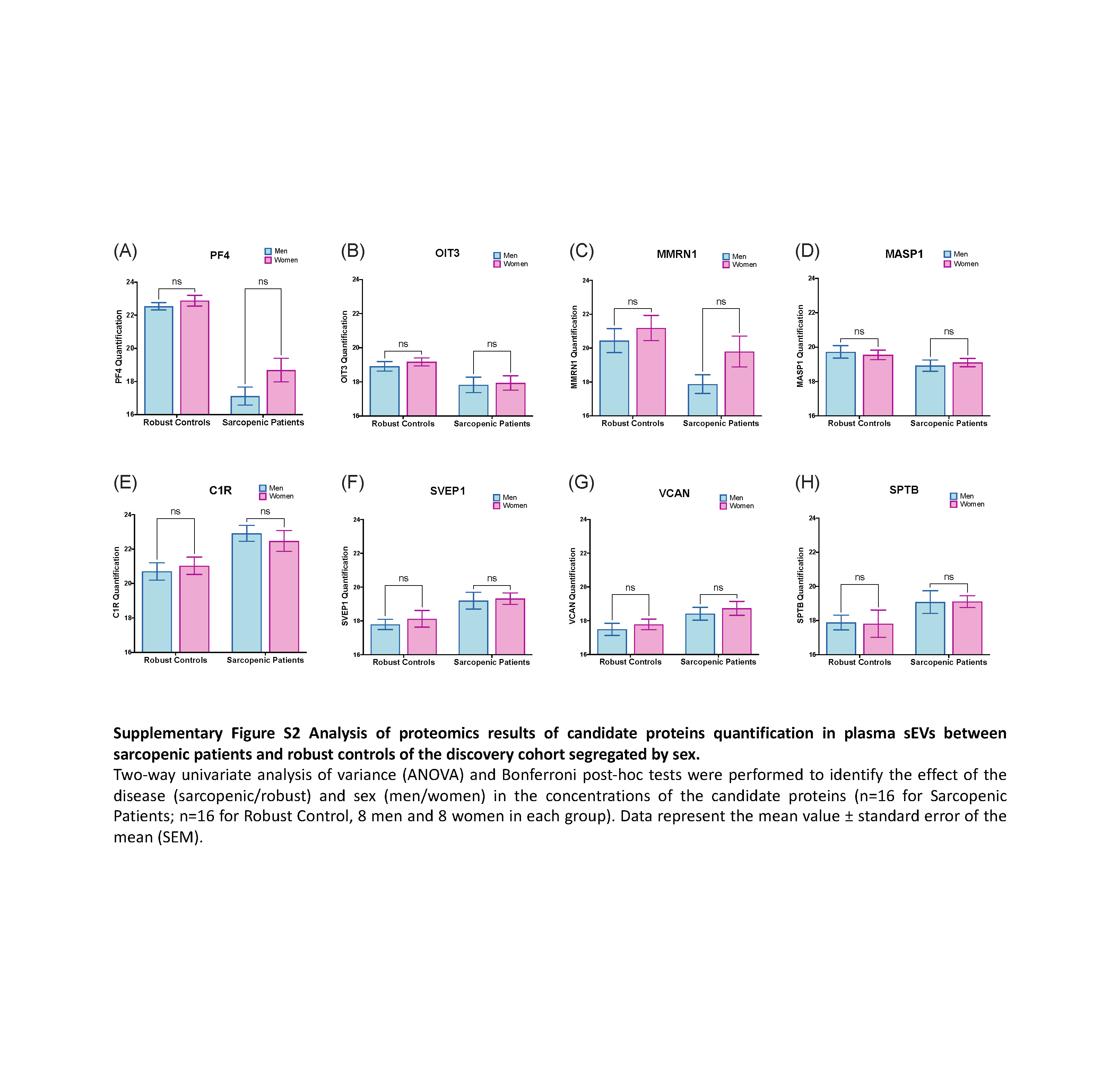

Supplement: Supplementary file 2 — Figure S2 Analysis of proteomics results of candidate proteins quantification in plasma sEVs between sarcopenic patients and robust controls of the discovery cohort segregated by sex. Two‐way univariate analysis of variance (ANOVA) and Bonferroni post‐hoc tests were performed to identify the effect of the disease (sarcopenic/robust) and sex (men/women) in the concentrations of the candidate proteins (n = 16 for Sarcopenic Patients; n = 16 for Robust Control, 8 men and 8 women in each group). Data represent the mean value ± standard error of the mean (SEM). [file JCSM-15-1883-s006.tiff]

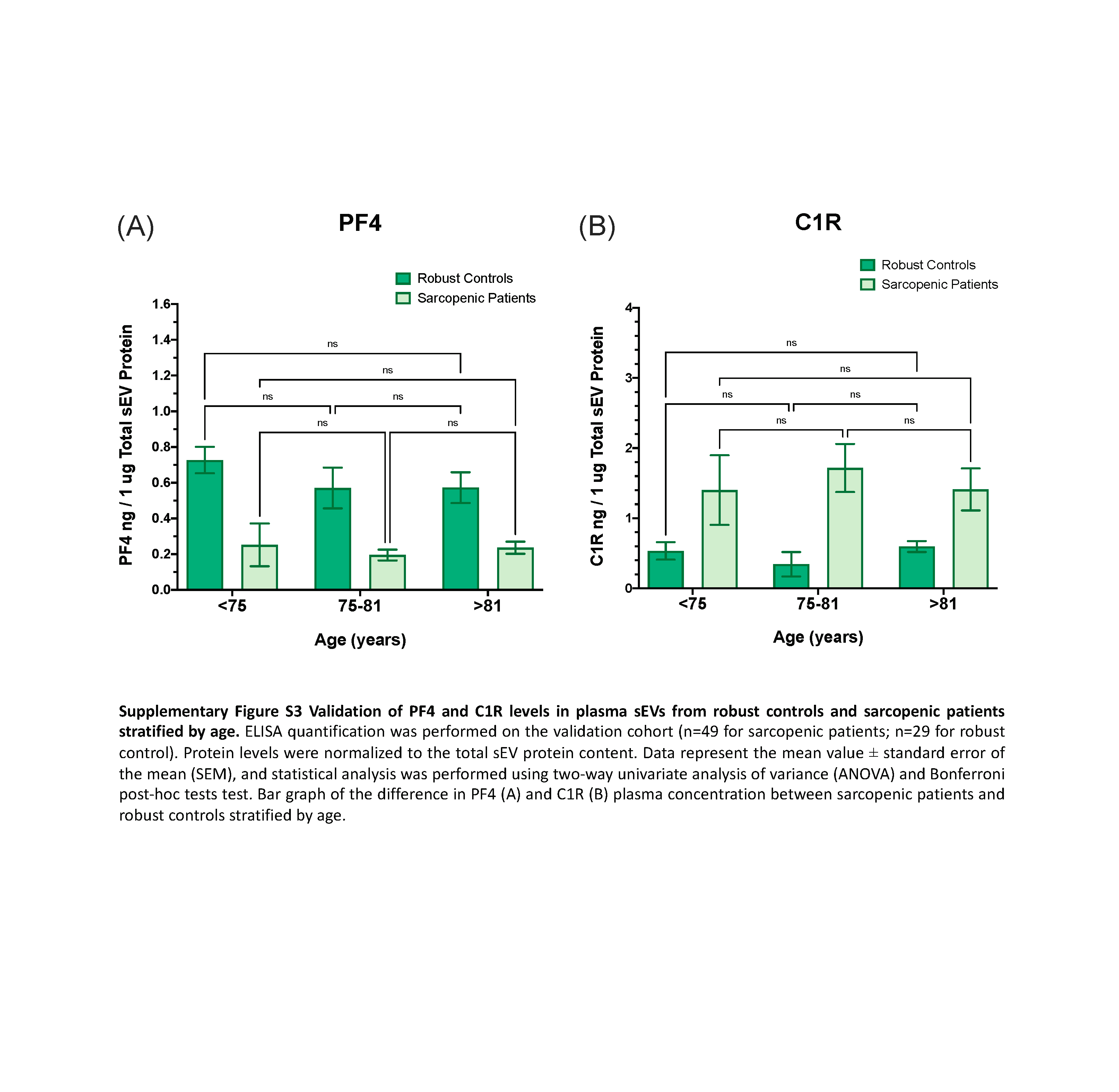

Supplement: Supplementary file 3 — Figure S3 Validation of PF4 and C1R levels in plasma sEVs from robust controls and sarcopenic patients stratified by age. ELISA quantification was performed on the validation cohort (n = 49 for sarcopenic patients; n = 29 for robust control). Protein levels were normalized to the total sEV protein content. Data represent the mean value ± standard error of the mean (SEM), and statistical analysis was performed using two‐way univariate analysis of variance (ANOVA) and Bonferroni post‐hoc tests test. Bar graph of the difference in PF4 (A) and C1R (B) plasma concentration between sarcopenic patients and robust controls stratified by age. [file JCSM-15-1883-s005.tiff]

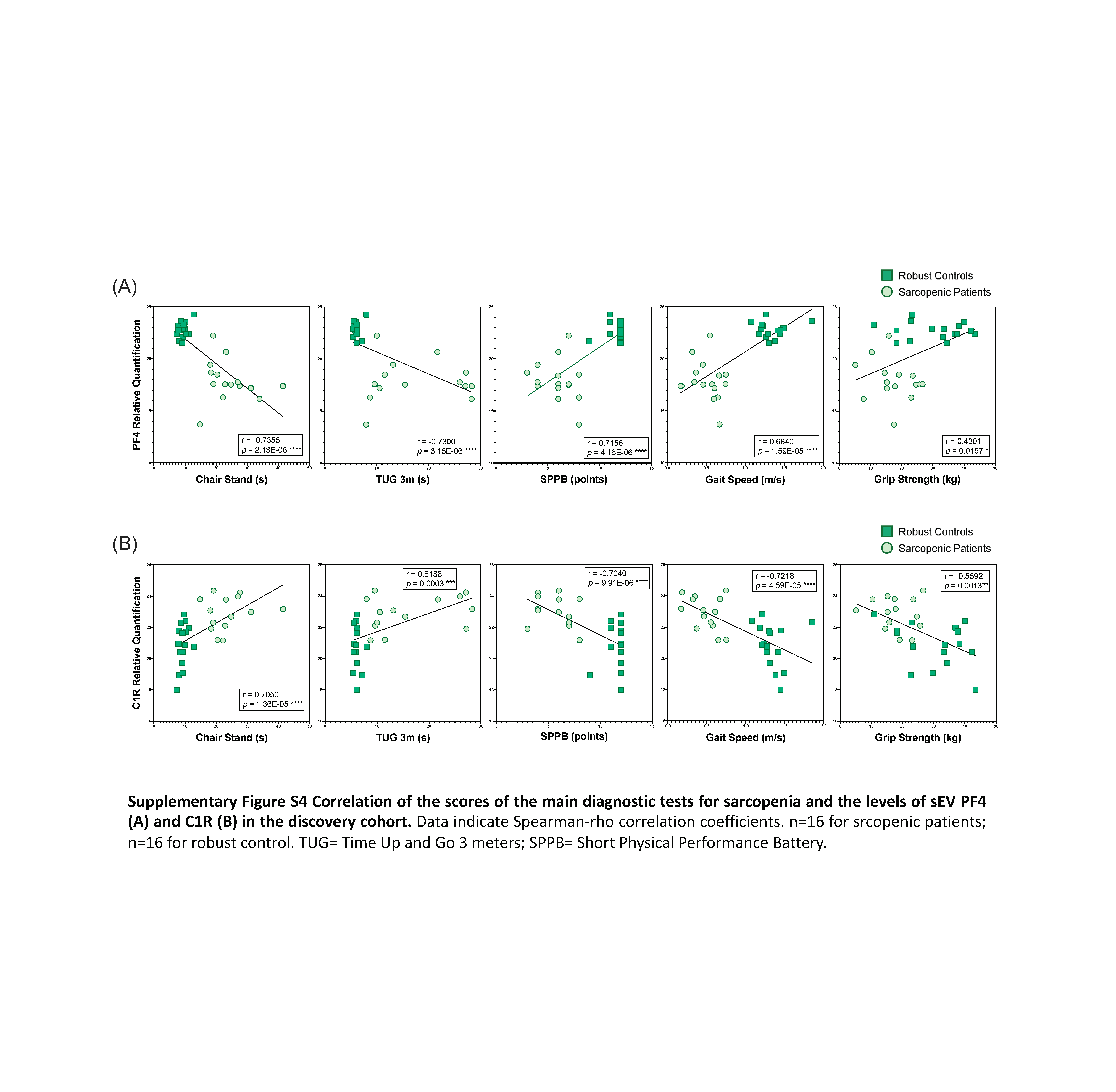

Supplement: Supplementary file 4 — Figure S4 Correlation of the scores of the main diagnostic tests for sarcopenia and the levels of sEV PF4 (A) and C1R (B) in the discovery cohort. Data indicate Spearman‐rho correlation coefficients. n = 16 for sarcopenic patients; n = 16 for robust control. TUG = Time Up and Go 3 meters; SPPB = Short Physical Performance Battery. [file JCSM-15-1883-s009.tiff]

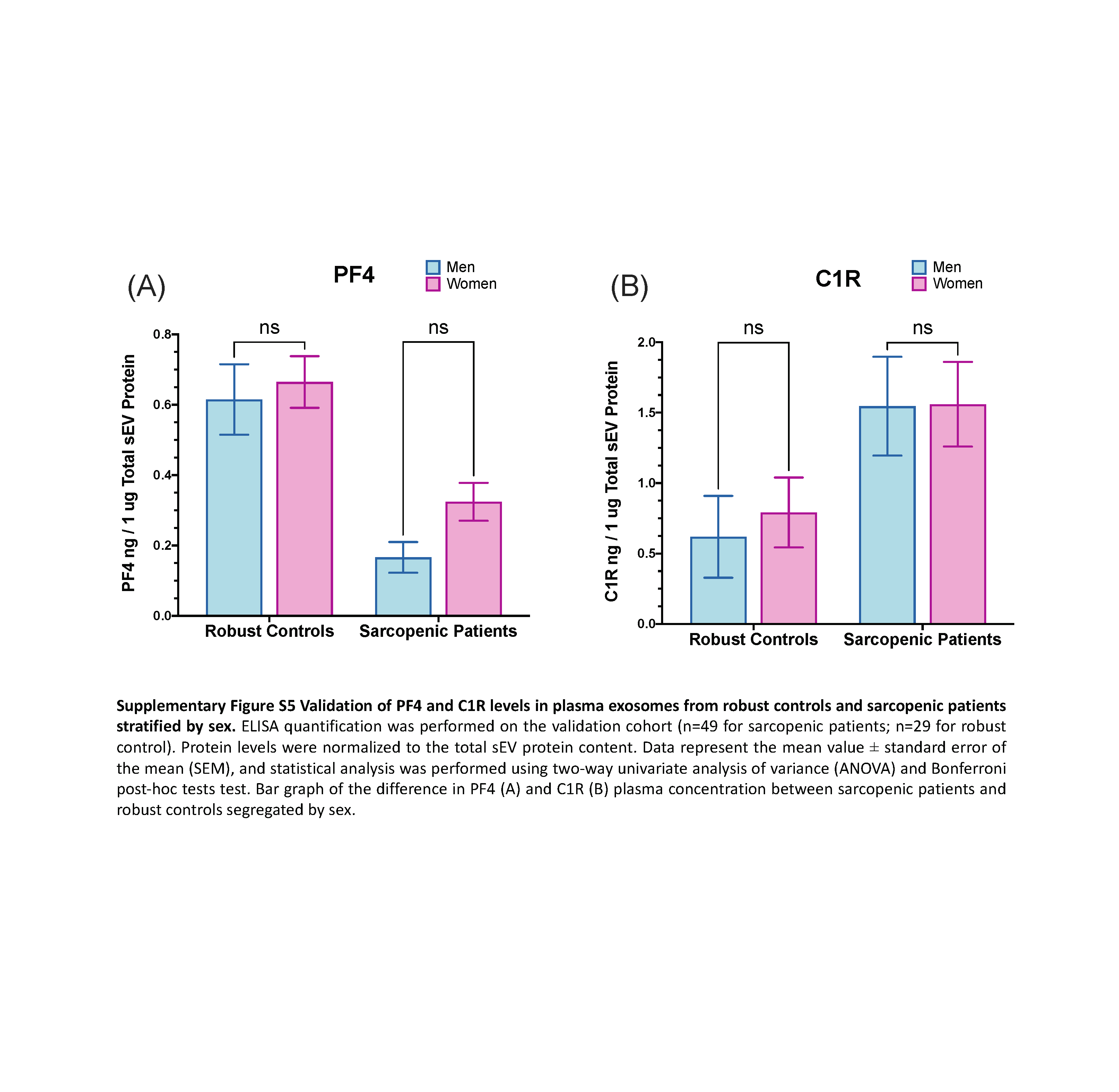

Supplement: Supplementary file 5 — Figure S5 Validation of PF4 and C1R levels in plasma exosomes from robust controls and sarcopenic patients stratified by sex. ELISA quantification was performed on the validation cohort (n = 49 for sarcopenic patients; n = 29 for robust control). Protein levels were normalized to the total sEV protein content. Data represent the mean value ± standard error of the mean (SEM), and statistical analysis was performed using two‐way univariate analysis of variance (ANOVA) and Bonferroni post‐hoc tests test. Bar graph of the difference in PF4 (A) and C1R (B) plasma concentration between sarcopenic patients and robust controls segregated by sex. [file JCSM-15-1883-s007.tiff]

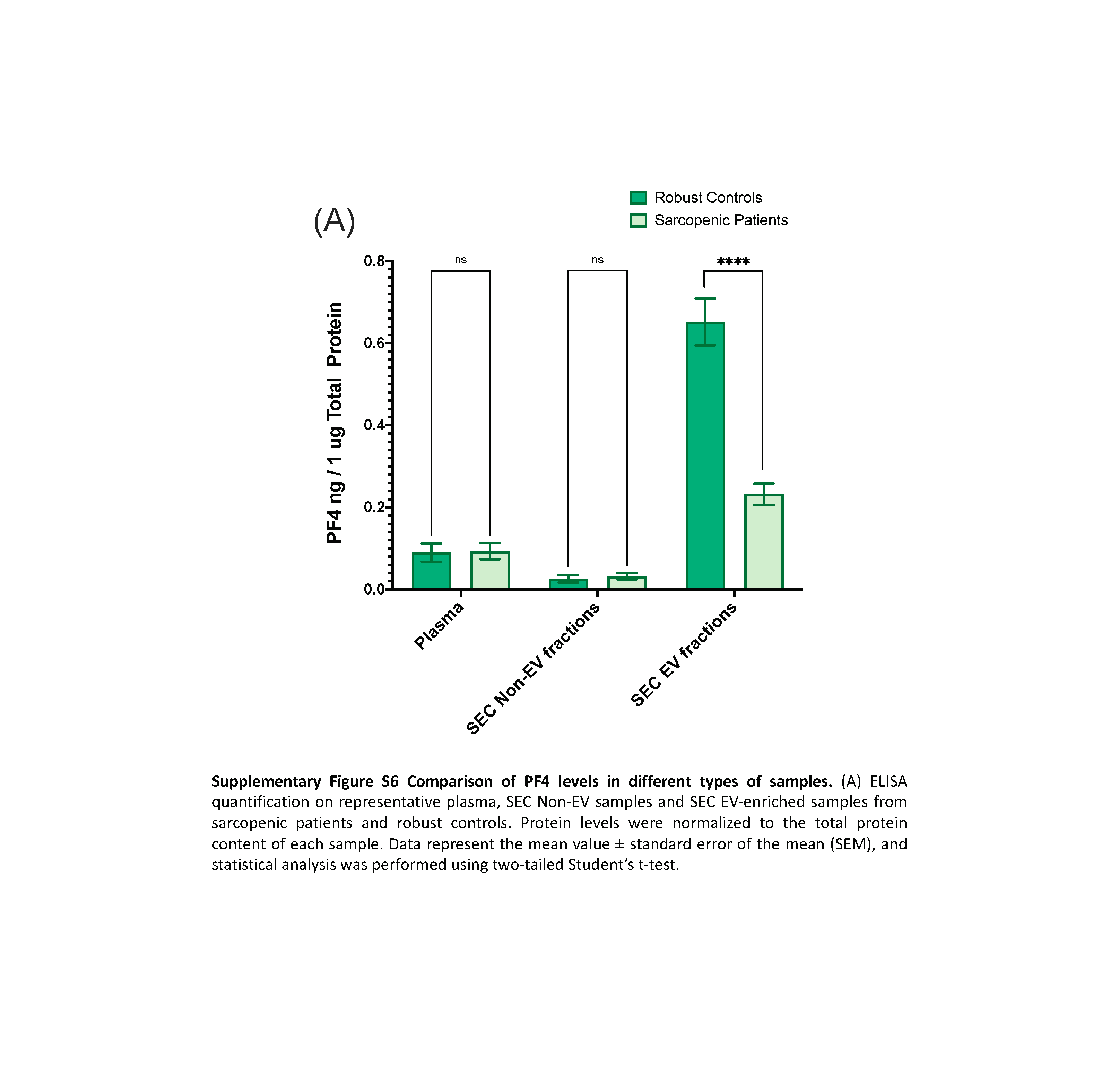

Supplement: Supplementary file 6 — Figure S6 Comparison of PF4 levels in different types of samples. (A) ELISA quantification on representative plasma, SEC Non‐EV samples and SEC EV‐enriched samples from sarcopenic patients and robust controls. Protein levels were normalized to the total protein content of each sample. Data represent the mean value ± standard error of the mean (SEM), and statistical analysis was performed using two‐tailed Student's t‐test. [file JCSM-15-1883-s002.tiff]

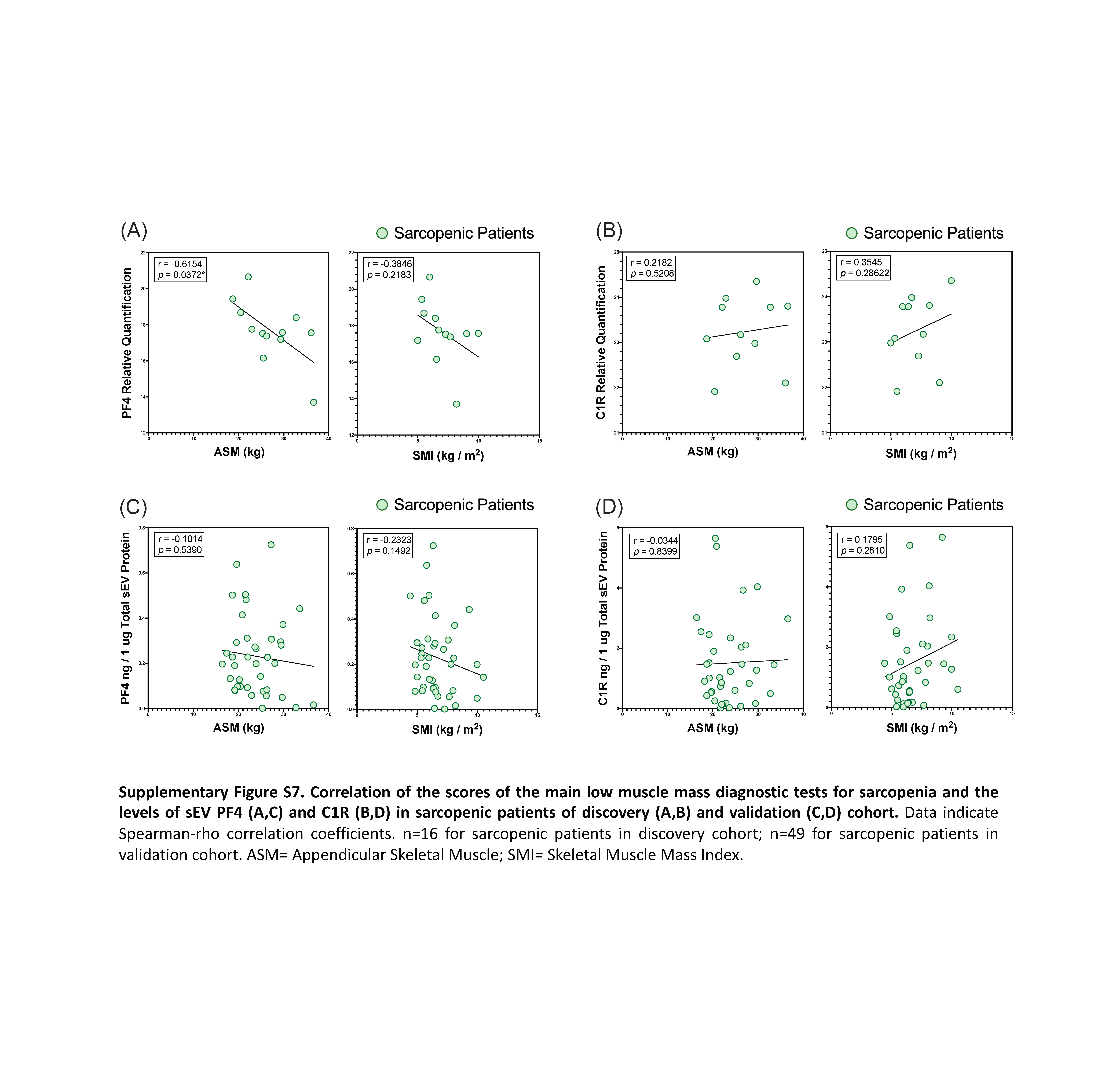

Supplement: Supplementary file 7 — Figure S7. Correlation of the scores of the main low muscle mass diagnostic tests for sarcopenia and the levels of sEV PF4 (A,C) and C1R (B,D) in sarcopenic patients of discovery (A,B) and validation (C,D) cohort. Data indicate Spearman‐rho correlation coefficients. n = 16 for sarcopenic patients in discovery cohort; n = 49 for sarcopenic patients in validation cohort. ASM = Appendicular Skeletal Muscle; SMI = Skeletal Muscle Mass Index. [file JCSM-15-1883-s004.tiff]
