# Supplementary material for: Proteomic profiling of human plasma extracellular vesicles identifies PF4 and C1R as novel biomarker in sarcopenia
Source: J Cachexia Sarcopenia Muscle. 2024 Jul 15;15(5):1883–97. doi: 10.1002/jcsm.13539 (PMC11446689; doi:10.1002/jcsm.13539)
Supplement: Supplementary file 8 — Data S1. Supporting Information. [file JCSM-15-1883-s010.pdf]

Supporting Information for the article:

Proteomic Profiling of Human Plasma Extracellular Vesicles Identifies PF4 and C1R as novel Biomarkers in Sarcopenia

Author(s)
Paula Aparicio 1,2,3, David Navarrete-Villanueva 3,4,5, Alba María Gómez Cabello 3,4,6, Tresa López-Royo 1,2,3, Enrique Santamaría 7, Joaquín Fernández-Irigoyen 7, Karina Ausín 7, Germán Vicente-Rodríguez 3,4,8,9, Rosario Osta* 1,2,3, Raquel Manzano* 1,2,3.
* Both authors contribute equally

Affiliation(s) and address(es) of the author(s)
(1) LAGENBIO, Faculty of Veterinary, University of Zaragoza, Miguel Servet 177, 50013 Zaragoza, Spain
(2) Centre for Biomedical Research in Neurodegenerative Diseases (CIBERNED), Instituto de Salud Carlos III, 28029 Madrid, Spain
(3) AgroFood Institute of Aragon (IA2), Institute of Health Research of Aragon (IIS), Zaragoza, Spain.
(4) GENUD (Growth, Exercise, Nutrition and Development) Research Group, University of Zaragoza, Zaragoza, Spain
(5) Faculty of Health Science, University of Zaragoza, Domingo Miral, s/n, 50009 Zaragoza, Spain
(6) Defense University Center, 50090 Zaragoza, Spain
(7) Proteomics Platform, Navarrabiomed, Hospital Universitario de Navarra (HUN), Universidad Pública de Navarra UPNA, IdiSNA, 31008 Pamplona, Spain
(8) Centro de Investigación Biomédica en Red de Fisiopatología de la Obesidad y Nutrición (CIBERObn)
(9) Faculty of Health and Sport Science (FCSD, Ronda Misericordia 5, 22001-Huesca, Spain), Department of Physiatry and Nursing, University of Zaragoza, Spain

Material and methods

2. ISOLATION AND PURIFICATION OF SMALL EXTRACELLULAR VESICLES FROM HUMAN PLASMA

Blood samples (3 mL) were obtained from all the participants in heparin-containing tubes, processed immediately as follows. 1.5 mL of PBS (Phosphate buffered saline) was added to each blood sample and density gradient centrifugation was performed at 800 g for 20 minutes using a LymphoprepTM (1856-4, Progen) cushion to separate plasma from PBMC (peripheral blood mononuclear cells)and erythrocytes.
Approximately, 2 mL of plasma was collected from the upper phase and stored at -80°C. Plasma samples were thawed at 4 °C. Size Exclusion Chromatography was performed following the manufacturer's protocol (PURE-EVs: Size exclusion chromatography columns, #HBM-PEV, HansaBiomed). First, the samples were subjected to a series of centrifugations (10 minutes at 300 g at 4°C; 20 minutes at 1,200 g at 4°C; 30 minutes at 10,000 g at 4°C) to remove broken cells and vesicles larger than 200 nm. After collecting the supernatant, 2 mL of each sample were deposited on the column and the collection of 24 sequential fractions of 0.5 mL immediately started with PBS as elution buffer. Fractions 1 to 6 and 12 to 24 were discarded and fractions 7 to 11, containing the highest amount of **EVs**, were pooled and saved. Pooled fractions were subjected to a two-step centrifugation in order to concentrate the **EVs** and further remove small plasma proteins and peptides improving sample purity. First, each fraction was transferred to a 300 kDa MWCO centrifugal concentrator (Vivaspin6 300 kDa MWCO, #VS0651, Sartorius) at 4,000 g for 6 minutes, at 4 °C. Then, it was centrifuged in a 10 kDa MWCO filter (Amicon Ultra-0.5, #UFC501096, Merck Millipore) at 14,000 g, for 10 minutes, at 4 °C. The supernatant containing the purified **EVs** was collected and stored at -80°C until further processed.

6. WESTERN BLOT (WB)

Supplementary infirmation: Primary and secondary antibodies used for Western Blot.

Antibody	Species of generation	Company	Reference	Dilution
Anti-CD81	M	BD Biosciences	555675	1:500
Anti-CD9	M	Santa Cruz Biotechnology	sc-59140	1:500

Anti-HSC70	G	Santa Cruz Biotechnology	sc-1059	1:500
**Anti-ALIX**	**M**	**Santa Cruz Biotechnology**	**sc-53540**	**1:500**
**Anti-CALNEXIN**	**R**	**Enzo Life Sciences**	**ADI-SPA-865**	**1:500**
Anti-mouse IgGκ BP-HRP	-	Santa Cruz Biotechnology	sc-516102	1:5,000
Anti-goat IgG-HRP	M	Santa Cruz Biotechnology	sc-2354	1:5,000
Anti-rabbit IgG-HRP	M	Santa Cruz Biotechnology	Sc-2357	1:5,000

Abbreviations: M, mouse; G, goat; R, rabbit.

7. PROTEIN IDENTIFICATION BY MASS SPECTROMETRY

Sample Preparation

Protein extracts of EV were diluted in Laemmli sample buffer and loaded into a 0.75 mm thick polyacrylamide gel with a 4% stacking gel casted over a 12.5% resolving gel. The run was stopped as soon as the front entered 3 mm into the resolving gel so that the whole proteome became concentrated in the stacking/resolving gel interface. Bands were stained with Coomassie Brilliant Blue, excised from the gel and protein enzymatic cleavage was carried out with trypsin (Promega; 1:20, w/w) at 37 °C for 16 h as previously described [S13]. Purification and concentration of peptides was performed using C18 Zip Tip Solid Phase Extraction (Millipore).

LC-MS/MS

Peptide mixtures were separated by reverse phase chromatography using an UltiMate 3000 UHLPC System (Thermo Scientific) fitted with an Aurora packed emitter column (Ionopticks, 25 cm x 75 µm ID, 1.6 µm C18). Samples were first loaded for desalting and concentration into an Acclaim PepMap column (ThermoFisher, 0,5 cm x 300 µm ID, 5 µm C18) packed with the same chemistry as the separating column. Mobile phases were 100% water 0.1% formic acid (FA) (buffer A) and 100% Acetonitrile 0.1% FA (buffer B). Column gradient was developed in a 120 min two step gradient from 5% B to 20% B in 90 min and 20%B to 32% B in 30 min. Column was equilibrated in 95% B for 10 min and 5% B for 20 min. During all process, precolumn was in line with column and flow maintained all along the gradient at 300 nl/min. The column temperature was maintained at 40 °C using an integrated column oven (PRSO-V2, Sonation, Biberach, Germany) and interfaced online with the Orbitrap Exploris 480 MS. Spray voltage were set to 2 kV, funnel RF level at 40, and heated capillary temperature at 300 °C. For DDA experiments full MS resolutions were set to 1,200,000 at m/z 200 and full MS AGC target was set to Standard with an IT mode Auto. Mass range was set to 375–1500. AGC target value for fragment spectra was set to Standard with a resolution of 15,000 and 3 seconds for cycle time. Intensity threshold was kept at 8E3. Isolation width was set at 1.4 m/z. Normalized collision energy was set at 30%. All data were acquired in centroid mode using positive polarity and peptide match was set to off, and isotope exclusion was on.

Data Analysis

Raw files were processed with MaxQuant [S14] v1.6.17.0 using the integrated Andromeda Search engine [S15]. All data were searched against a target/decoy version of the human Uniprot Reference Proteome with March 2021 release. First search peptide tolerance was set to 20 ppm, main search peptide tolerance was set to 4.5 ppm. Fragment mass tolerance was set to 20 ppm. Trypsin was specified as enzyme, cleaving after all lysine and arginine residues and allowing up to two missed cleavages. Carbamidomethylation of cysteine was specified as fixed modification and peptide N-terminal acetylation, oxidation of methionine, deamidation of asparagine and glutamine and pyro-glutamate formation from glutamine and glutamate were considered variable modifications with a total of 2 variable modifications per peptide. "Maximum peptide mass" were set to 7500 Da, the "modified peptide minimum score" and "unmodified peptide minimum score" were set to 25 and everything else was set to the default values, including the false discovery rate limit of 1% on both the peptide and protein levels. Proteins with more than 2 missed cleavages, a false discovery rate of >1% or less than 3 unique peptides were excluded. The Perseus software (version 1.6.14.0) [S16] was used for statistical analysis and data visualization. Two-way univariate analysis of variance (ANOVA) and Bonferroni post-hoc tests were performed to identify the effect of the disease (sarcopenic/robust) and sex (men/women) in the concentrations of these proteins. Among the proteins identified those with a p value less than 0.05 and a Log2 fold change greater than 0.5 or less than -0.5 in the statistical analysis when comparing the sarcopenic and robust group were considered to be differentially regulated.

Additional references

[S1] Ethgen O, Beaudart C, Buckinx F, Bruyère O, Reginster JY. The Future Prevalence of Sarcopenia in Europe: A Claim for Public Health Action. Calcif Tissue Int 2017;100:229–34.

[S2] Théry C, Zitvogel L, Amigorena S. Exosomes: composition, biogenesis and function. Nat Rev Immunol 2002;2:569–79.

[S3] McKiernan J, Donovan MJ, O'Neill V, Bentink S, Noerholm M, Belzer S, et al. A Novel Urine Exosome Gene Expression Assay to Predict High-grade Prostate Cancer at Initial Biopsy. JAMA Oncol 2016;2:882.

[S4] Brinkmann K, Enderle D, Koestler T, Bentink S, Emenegger J, Spiel A, et al. Abstract 545: Plasma-based diagnostics for detection of EML4-ALK fusion transcripts in NSCLC patients. Cancer Res 2015;75:545–545.

[S5] Perracini MR, Mello M, de Oliveira Máximo R, Bilton TL, Ferriolli E, Lustosa LP, et al. Diagnostic Accuracy of the Short Physical Performance Battery for Detecting Frailty in Older People .Vol. 100, Physical Therapy. Oxford University Press (OUP); 2019. p. 90–8.

[S6] Janssen I. Skeletal Muscle Cutpoints Associated with Elevated Physical Disability Risk in Older Men and Women. Vol. 159, American Journal of Epidemiology. Oxford University Press (OUP); 2004. p. 413–21.

[S7] Szklarczyk D, Gable AL, Lyon D, Junge A, Wyder S, Huerta-Cepas J, et al. STRING v11: protein-protein association networks with increased coverage, supporting functional discovery in genome-wide experimental datasets. Nucleic Acids Res 2019;8;47(D1):D607-D613.

[S8] Keerthikumar S, Chisanga D, Ariyaratne D, Al Saffar H, Anand S, Zhao K, et al. ExoCarta: A Web-Based Compendium of Exosomal Cargo. J Mol Biol 2016;428:688–92.

[S9] Pathan M, Fonseka P, Chitti S V, Kang T, Sanwlani R, Van Deun J, et al. Vesiclepedia 2019: a compendium of RNA, proteins, lipids and metabolites in extracellular vesicles. Nucleic Acids Res 2019;47:D516–9.

[S10] Nahm FS. Receiver operating characteristic curve: overview and practical use for clinicians. Korean J Anesthesiol 2022;75:25–36.

[S11] Reggiani C, Schiaffino S. Muscle hypertrophy and muscle strength: dependent or independent variables? A provocative review. Vol. 30, European Journal of Translational Myology. PAGEPress Publications; 2020.

[S12] Riviati N, Indra B. Relationship between muscle mass and muscle strength with physical performance in older adults: A systematic review. Vol. 11, SAGE Open Medicine. SAGE Publications; 2023.

[S13] Shevchenko A, Tomas H, Havli J, Olsen J V, Mann M. In-gel digestion for mass spectrometric characterization of proteins and proteomes. Nat Protoc 2006;1:2856–60.

[S14] Cox J, Mann M. MaxQuant enables high peptide identification rates, individualized p.p.b.-range mass accuracies and proteome-wide protein quantification. Nat Biotechnol 2008;26:1367–72.

[S15] Cox J, Neuhauser N, Michalski A, Scheltema RA, Olsen J V., Mann M. Andromeda: A Peptide Search Engine Integrated into the MaxQuant Environment. J Proteome Res 2011;10:1794–805.

[S16] Tyanova S, Temu T, Sinitcyn P, Carlson A, Hein MY, Geiger T, et al. The Perseus computational platform for comprehensive analysis of (prote)omics data. Nat Methods 2016;13:731–40.
