# Supplementary material for: Proteomic profiling of human plasma extracellular vesicles identifies PF4 and C1R as novel biomarker in sarcopenia
Source: J Cachexia Sarcopenia Muscle. 2024 Jul 15;15(5):1883–97. doi: 10.1002/jcsm.13539 (PMC11446689; doi:10.1002/jcsm.13539)
Supplement: Supplementary file 10 — Data S2. Supporting Information. [file JCSM-15-1883-s001.docx]

**CRediT authorship contribution statement**

P.A.: Conceptualization, Methodology, Validation, Formal Analysis, Writing – original draft; D.N.V.: Methodology, Writing – review & editing; A.G.C.: Methodology; T.L.R.: Methodology, Writing – review & editing; E.S.: Supervision; J.F.I.: Methodology; K.A.: Methodology; M.A.: Methodology, Writing – review & editing; V.S.: Methodology, Writing – review & editing; G.V.R.: Conceptualization, [Supervision](http://159.203.176.220/contributor-roles/supervision/), [Funding acquisition](http://159.203.176.220/contributor-roles/funding-acquisition/); R.O.: Conceptualization, [Supervision](http://159.203.176.220/contributor-roles/supervision/), [Funding acquisition](http://159.203.176.220/contributor-roles/funding-acquisition/), Writing – review & editing; R.M.: Conceptualization, [Supervision](http://159.203.176.220/contributor-roles/supervision/), [Funding acquisition](http://159.203.176.220/contributor-roles/funding-acquisition/), Writing – review & editing. All the authors read and approved the submitted version.
